# Infections of *Aedes* Mosquito Cells by *Wolbachia* Strains *w*Au and *w*Melpop Modulate Host Cellular Transcriptomes Differently and Suppress Dengue Viral Replication

**DOI:** 10.3390/v17070922

**Published:** 2025-06-28

**Authors:** Amber R. Mickelson, Julia Felton, Olivia Cheschi, Emily Spacone, Kaitlyn Connors, Jacob Thornsberry, Tadahisa Teramoto

**Affiliations:** Department of Microbiology and Immunology, Georgetown University, Washington, DC 20057, USA; arm376@georgetown.edu (A.R.M.); jaf379@georgetown.edu (J.F.); omc16@georgetown.edu (O.C.); ems405@georgetown.edu (E.S.); kc1430@georgetown.edu (K.C.); jacob.thornsberry@louisville.edu (J.T.)

**Keywords:** World Mosquito Program, *Wolbachia*, *w*MelPop, *w*Au, *Aedes* mosquitoes, dengue virus, RNA-seq

## Abstract

Dengue virus serotypes 1-4 (DENV1-4) have spread through tropical and subtropical countries, causing endemic and epidemic diseases. Recently, a novel field approach using the *Wolbachia* symbiont was proposed to suppress DENV transmission via the mosquito vectors *Aedes aegypti* and *Aedes albopictus*. Previously, we showed that a *Wolbachia* strain, *w*MelPop, suppresses DENV2 replication in the C6/36 *albopictus* cell line, with the mutant DENV2 appearing and replacing the wild type DENV2. In this study, we expanded the analysis to include replications of all DENV serotypes 1-4, effects of *w*Au *Wolbachia* in C6/36 cells, and *w*MelPop-influences on the Aag2 *aegypti* cell line. It was revealed that both *w*Au *and w*MelPop reduce all DENV infectious titers without dominant appearances of the mutant viruses, despite varied effects on the viral copy numbers. The host transcriptomic profiles by RNA-seq were also variously altered by *w*Au and *w*MelPop (ranging from 10 to 30%, Log_2_FC > 2 or <−2, *p* < 0.05). Those transcripts were not further altered by DENV infection. In contrast, abundant transcriptomic alterations by DENV infection in naïve C6/36 and Aag2 cells were blocked by either *w*Au *or w*MelPop. These results indicate that *Wolbachia* prevents host cellular transcriptomic alterations which are induced by DENV infection, affecting the cellular homeostasis necessary for DENV replication.

## 1. Introduction

*Wolbachia pipentis* bacteria are intracellular symbionts that were first discovered in *Culex pipentis* mosquitoes [1] and have been found in many invertebrates, mainly arthropods and filarial nematodes [2,3]. Two-thirds of all insect species are estimated to carry *Wolbachia* [4]. *Wolbachia pipentis* provides reproductive advantages to the infected females by manipulating fertilization, such as causing cytoplasmic incompatibility (CI), by which *Wolbachia*-infected sperms lead to embryo death [5]. CI contributes to the selective advantages of *Wolbachia* in maternal inheritance, spreading through host populations. The mechanisms of CI and its rescue in embryos have been greatly analyzed (review in [6]). There are also *Wolbachia* strains that do not induce CI. It was reported that *w*Au infecting *Drosophila simulants* does not cause CI [7]. The *w*MelPop strain also does not induce CI in its native host, *Drosophila melanogaster*. However, it heavily infects somatic tissues, specifically neurons and muscle tissues, inducing tissue degeneration with popcorn-like vacuoles and reducing the fly’s lifespan [8]. Moreover, artificial transinfection of *w*MelPop into other species, such as *Drosophila simulans* and *Aedes (Ae.) aegypti* mosquitoes, induced CI in addition to these somatic tissue damages [9,10]. In contrast, transinfections of *w*Au into other species have not induced CI, or deleterious tissue damage [11]. *Wolbachia* are mutualists, providing benefits to the hosts, such as essential metabolites [12,13,14] and protection against pathogens, such as RNA viruses and parasites [11,15,16,17]. It is considered that these advantages are factors that have advanced *Wolbachia’s* spread into the host species. The efficiency of *Wolbachia*’s host-infiltration also depends on a higher maternal transmission, by which a genetically close, avirulent strain, *w*Mel, could fully spread to *Ae. aegypti* populations within fewer generations, compared to a pathogenic *w*MelPop [18].

Mosquitoes transmit various pathogens to humans. Many of these are circulating within zoonotic cycles. Therefore, infections to humans are incidental. However, the four serotypes of dengue virus (DENV1-4), members of the *Flavivirus* genus in the *Flaviviridae* family, transmitted by *Ae. aegypti* and *Ae. albopictus*, have established a human–mosquito transmission cycle. Dengue virus infections cause endemic and epidemic diseases throughout tropical and subtropical regions. High fever and flu-like symptoms are mostly resolved in 7–10 days and confer a life-long immunity against the infected serotype. However, the symptoms can progress to fatal complications, known as severe dengue, accompanied by liver failure and/or circulatory shock, especially with secondary infections by a different DENV serotype under the antibody-dependent enhancement (ADE) mechanism [19]. Severe dengue symptoms lead to ~25,000 deaths annually [20]. There are no antiviral drugs for treating DENV infections. For prevention, there is one FDA-approved dengue vaccine (Dengvaxia), that consists of tetravalent live attenuated viral strains. However, its usage is limited to individuals aged 6–16 who have experienced a previous laboratory-confirmed DENV infection by one serotype. The age group less than 6 years old among vaccinated children showed increased hospitalization by the DENV infection [21]. It is considered that vaccine-induced ADE causes the severity of the infection in those children [21,22].

The effects of *Wolbachia* in protecting insects from their pathogens have been variously reported. Irrespective of CI or tissue damage by *Wolbachia*, wMel, *w*MelPop, and *w*Au strains were shown to suppress transmissions of pathogens, including flaviviruses, such as DENV and West Nile virus (WNV), and alphaviruses, such as Chikungunya virus (CHIKV), in addition to malarial parasites [10,18,23,24,25,26,27,28]. These effects have led to promising field studies for releasing *w*Mel- or *w*MelPop-transinfected *Ae. aegypti* by the World Mosquito Program (WMP) led by Dr. O’Neill’s group in Australia [29,30,31,32]. Lowered DENV cases in these mosquito-released areas have been reported [33,34].

DENV genome is a single-stranded, ~11 kb-length, positive-sense RNA, encoding a single open reading frame flanked by 5′- and 3′-untranslated regions (UTR), with 5′-cap but lacking 3′-poly(A) tail, from which a single polyprotein is translated [35,36]. The polyprotein is cleaved by both cellular and viral proteases into three structural proteins, (capsid [C], precursor membrane [prM], and envelope [E]), and seven non-structural (NS) proteins (NS1, NS2A, NS2B, NS3, NS4A, NS4B, and NS5) [35]. The NS1 to NS5 proteins, together with known/unknown host proteins, collaborate to reproduce infectious DENV particles.

In this study, we observed that *w*Au and *w*MelPop similarly suppressed DENV1-4 replication and/or infectivity in C6/36 cells, *Ae. albopictus-*derived cell lines. *w*MelPop also suppressed DENV1-4 replication/infectivity in Aag2 cells, an *Ae. aepypti-*derived cell line. It was revealed that cellular transcriptomes in C6/36 cells were affected differently by high- or low-density *w*Au or *w*MelPop presence in the cells. Transcripts in Aag2 cells and C6/36 cells also responded differently to *w*MelPop. DENV2 infection induced transcriptome and cellular pathway alterations in naïve cells, although these changes were blocked by either *w*Au or *w*MelPop in both C6/36 cells and Aag2 cells. These results indicate that *Wolbachia* prevents cellular transcriptomic alterations which are induced by DENV infection and are necessary for DENV replication.

## 2. Materials and Methods

**Mosquito cell line culture and authentication of *Wolbachia* containment.** The endosymbionts *w*MelPop- or *w*Au-transinfected C6/36 (*Ae. albopictus)* cells (referred to here as *w*MelPop- or *w*Au-C6/36 cells) as well as *w*MelPop-Aag2 *(Ae. aegypti*) cells were established in Dr. ONeill’s laboratory at Monash University in Australia and were provided for this study [37]. All these cells were cultured in minimum Dulbecco’s modified Eagle medium (DMEM) containing 5% FBS, 2 mM L-glutamine, 25 mM HEPES, pH 7.2, 1 mM sodium pyruvate, 1× non-essential amino acids, 100 U/mL penicillin, and 100 μg/mL streptomycin at 30 °C. The naïve C6/36 cells were obtained from the American Type Culture Collection (ATCC), while naïve Aag2 cells were developed by tetracyclin treatment (10 μg/mL) over 3 successive passages to eradicate *w*MelPop [37]. Cellular DNAs were extracted using the digested solution containing 10 mM Tris-HCl, pH 8.0, 100 mM NaCl, 25 mM EDTA, 0.5% (*w*/*v*) SDS, and 0.1 mg/mL proteinase K, followed by phenol/chloroform/isoamyl alcohol extraction and ethanol precipitation as described [38]. These cells with or without *Wolbachia* were authenticated by PCR. The *Wolbachia* copy numbers per cell were calculated by qPCR. The *Wolbachia* gene 16S ribosomal (r) RNA was targeted to detect both *w*MelPop and *w*Au, while the “catalase” gene, which is localized at a mosquito nuclear genome, was chosen to determine cell numbers in the samples. We selected the common sequence regions of 16S rRNA and catalase genes in both *w*MelPop and *w*Au as well as *Ae. albopictus* and *Ae. aegypti*, to anneal the identical DNA sequences in different species (the gene sequences and the primer oligo-annealing sequences are shown in the Supplemental Method File). PCR product sizes targeting 16S r RNA and the catalase genes were 160 bp and 184 bp, respectively (Supplemental Method File). qPCR was performed with iTaq Universal SYBR super mix (Bio-Rad, Hercules, CA, USA) in a MIC qPCR cycler (Bio Molecular Systems, Upper Coomera, Australia) for 40 cycles, each consisting of 95 °C denaturation for 5 s, 60 °C annealing for 20 s, and 72 °C extension for 10 s. The gene copy numbers in the samples were calculated by comparing the threshold cycle (Ct) values of those samples with the prepared standard amounts of the genes.

We followed the report from Khoo et al. on the cell culture conditions to increase the *Wolbachia* infection into cells [39]. We kept higher cell densities with longer culturing times by delaying the trypsin-used splitting interval from 4 to 8 days. This simple method was continued for a year (i.e., beyond 40 passages).

**In situ hybridization to detect *Wolbachia* in the cultured cells.** Cells seeded in 8-well chambered coverslips (ibid, Germany) were washed with PBS and fixed with 4% paraformaldehyde at 4 °C for 30 min, followed by 0.1 M phosphate buffer washing 3 times. The fixed cells were sequentially dehydrated by 70%, 95%, and 100% *v*/*v* ethanol/water at room temperature. The DNA oligo nucleotide labelled at the 5′ terminus with Cy3 fluorescent dye, 5′-/5Cy3/ CTTCTGTGAGTACCGTCATTATC (IDT), was used as a probe for detecting 16S rRNA in *w*MelPop and *w*Au (Supplemental Method File). In situ hybridization was performed at 37 °C for 3 h with the DNA-oligo solution (10 ng/μL), containing 50% deionized formamide *v*/*v*, 4× SSC, 0.5× Denhardt’s, and 0.1 M DTT. Then, slides were sequentially rinsed at 55 °C for 15 min with 1× SSC buffer containing 10 mM DTT, followed by twice with 0.5× SSC buffer containing 10 mM DTT. The cells were stained with DAPI solution (2.5 μg/mL, Thermo Scientific, Waltham, MA, USA) and washed 3 times by PBS. A Leica SP8 laser scanning confocal microscope was used for visualizing the fluorescent staining.

**DENV1-4 infections. The** BHK21 cell line (a cell line of baby hamster kidney, purchased from ATCC) was transfected with DENV1, DENV2, or DENV4 RNAs, which was in vitro SP6 RNA polymerase-transcribed from the individual cDNA-containing plasmids provided by Dr. Falgout, FDA, Maryland. Amplified DENV3 was also provided by Dr. Falgout. Each DENV-containing supernatant was used to infect naïve C6/36 cells to further increase virus titers, which were confirmed as ~10^6^ PFU/mL by plaque assays using LLC/MK2 cells (a cell line of rhesus monkey kidney, purchased from ATCC) as described below [40]. These prepared DENV1-4 stock solutions were diluted into 70% confluent monolayer on the next day after splitting cells (cell numbers were estimated from doubling time as 20–24 h for both cells) for infections to *Wolbachia*-present or absent C6/36 or Aag2 cells at MOI of 1. Five days post-infection, supernatants in cell cultures were collected at 3000 rpm for 5 min. Cultured cells were also used for RNA extraction.

**Measurement of virus infectious titers by plaque assay.** LLC/MK2 cells were cultured in DMEM containing 2 mM L-glutamine,100 U/mL penicillin, 100 μg/mL streptomycin, and 5% FBS at 37 °C in a 5% CO_2_ incubator. Supernatants collected from DENV-infected cells were inoculated into LLC/MK2 cells seeded in duplicated wells in 6-well plates by diluting to 10^3^, 10^4^, and 10^5^. After 2 h of incubation at 37 °C, the medium was replaced with the solid medium containing 1% nonessential amino acids and 0.9% SeaPlaque^TM^ agarose (Lonza, Basel, Switzerland). After the medium solidified at room temperature, plates were incubated at 37 °C. Incubation time was varied based on the speeds of the viral spread causing cytotoxicity; DENV1: 11 days, DENV2: 15 days, DENV3: 9 days, DENV4: 13 days (plaque sizes enlarged time-dependently). These incubation times were determined by the days to distinguish the plaques. After the indicated incubation time had passed, the cells in the plates were fixed with 2 mL of formalin. The plaques were visualized by staining with 0.01% crystal violet solution. The plaque numbers in duplicated wells were counted.

**RNA extractions and RT-qPCR.** Viral RNAs in supernatants of infected cells were collected after 300 g centrifugation and extracted using a Quick-RNA^TM^-viral kit (Zymo Research, Irvine, CA, USA), while cellular RNA extractions were performed with Trizol (Thermo Fisher Scientific, according to the manufacturer’s instructions). From these RNAs, cDNAs were made by reverse transcriptase (RT) reactions at 42 °C for 2 h with ProtoScript II RT, random primers, and dNTPs (New England Biolabs). To quantify DENV1-4 RNA copy numbers, RT-qPCR was performed in duplicate with each DENV serotype-specific primer pair (Supplemental Table File) in the fluorescently labelled nucleotide-containing qPCR buffer and the cycler apparatus as described above. The acquired Ct values were converted to viral copy numbers by comparing them with the standardized concentrated individual DENV cDNA.

**RNA-seq library preparation, sequencing, and data collection to fastq files.** Biological triplicates of each experimental condition and treatment were prepared for RNAseq: (1) naïve C6/36 cells, (2) naïve C6/36 cells infected with DENV2, (3) *w*MelPop-C6/36 cells, (4) *w*MelPop-C6/36 infected with DENV2, (5) low-density *w*Au-C6/36 cells, (6) high-density *w*Au-C6/36 cells, (7) low-density *w*Au-C6/36 cells infected with DENV2, (8) high-density *w*Au-C6/36 cells infected with DENV2, (9) naïve Aag2 cells, (10) naïve Aag2 cells infected with DENV2, (11) *w*MelPop-Aag2 cells, and (12) *w*MelPop-Aag2 cells infected with DENV2. Cellular RNA quantity and integrity were determined on an Agilent 2100 Bioanalyzer (Agilent Technologies, Santa Clara, CA, USA). Illumina paired-end mRNA-Seq library construction and RNA-seq sequencing were performed at Zymo Research Inc. (Irvine, CA, USA). Using the Zymo-Seq RiboFree Total RNA Library kit (Zymo Research, Irvine, CA, USA), each RNA sample was purified and was reverse-transcribed to cDNAs, which was then ligated with the P7 adaptor sequence at the 3′ end, followed by second-strand synthesis and P5 adaptor ligation to the opposite sites of the double-stranded DNAs. After purification, based on DNA size (300–600 bp) with beads in the kit, index PCR was performed (according to the manufacturer’s protocol). Successful libraries were confirmed with Agilent’s D1000 Screen Tape Assay (Agilent Technologies, Santa Clara, CA, USA) on TapeStation and were sequenced on an Illumina Novaseq to a sequence depth of >30 million read pairs (150 bp paired-end sequencing). Individual paired read data (R1 and R2) were collected in separate fastq files.

**RNA-seq transcriptome analysis.** Read mapping, transcript assembly, and differential gene expression analyses were performed using Geneious Prime (Biomatters, v. 2024.7.0, Auckland, New Zealand). Geneious is a rare tool used for differential gene expression analysis but efficiently combines multiple genome and gene expression analysis tools. It has been used in several gene expression analysis studies [41,42,43]. Sequence reads in fastq files (R1 and R2) were set as a paired end and mapped to the annotated *Ae. albopictus* genome (https://www.ncbi.nlm.nih.gov/datasets/genome/GCF_035046485.1/ (accessed on 10 January 2023) or the *Ae. aegypti* genome (https://www.ncbi.nlm.nih.gov/datasets/genome/GCF_002204515.2/ (accessed on 10 January 2023) using the “Map to Reference” function. Transcript counts were obtained using the “Calculate Expression Levels” function. Normalized TPM (transcript per million) counts were generated, based on the differences in the total numbers of mRNAs. Differential expression Log_2_ fold-change ratios were determined using the “Compare Expression Levels” function. Differentially expressed genes were identified as having an absolute value of Log_2_ fold-change greater than 2 or less than −2 with a *p*-value less than 0.05. Volcano plots were generated using the Log_2_ fold change value and *p*-value on the SRplot website (https://www.bioinformatics.com.cn/srplot (accessed on 4 January 2024)) [44]. Venn diagrams were created using the website jvenn (https://jvenn.toulouse.inrae.fr (accessed on 4 January 2024)) [45]. Genes with Log_2_DER values were input to StringDB (https://string-db.org (accessed on 4 January 2024)) to calculate the enriched Gene Ontology molecular functions, biological processes, and cellular components. Up- (Log_2_DER > 0) and downregulated (Log_2_DER < 0) genes were analyzed separately to identify up- and downregulated pathways as suggested by Hong et al. [46]. GO Pathways were visualized as a Bubble Diagram constructed on the SRplot website.

**RNA-seq alignment analysis of DENV2 sequences.** The fastq files described above were also used for analysis of frequencies and locations of nucleotide changes in DENV RNA using Geneious software (Biomatters, v. 2024.7.0, Auckland, New Zealand) as previously described [47]. In addition, viral genomes in the extracellular particles were analyzed. Supernatants (~10 mL) from cell cultures in T-75 flasks were centrifuged at 15,000× *g* for 45 min in 40% PEG8000 solution containing 10 mM Tris (pH 8.0), 120 mM NaCl, and 1 mM EDTA [47]. Viral particles in the pellets and the remaining 0.5 mL solutions were thoroughly mixed with 1.5 mL Trizol-LS (Ambion, Austin, TX, USA) and 0.4 mL chloroform. After the centrifugation at 12,000× *g* for 15 min, the upper layer that separated from Trizol/chloroform (bottom layer) was used for ethanol precipitation to collect viral RNA. The pellet was washed with 70% ethanol, dried, and dissolved by Tris (10 mM, pH8.0)-buffered DEPC-water. The RNA was selected with the RNA Clean & Concentrator^TM^ kit (Zymo Research, Irvine, CA, USA), containing DNase. Library preparation and RNA-seq sequencing were performed at Zymo Research Inc. (Irvine, CA, USA), mentioned above. Alignment analyses of DENV sequences using Geneious Prime software (Biomatters, v. 2024.7.0, Auckland, New Zealand) were performed after trimming the 3′ end of R1 and R2 by the BBDuk plugin, from which the paired-end sequence data were made and aligned to the reference sequence. The created contigs were analyzed for SNPs. Nucleotide alterations with more than 5% frequency were listed (Supplemental Table File).

## 3. Results

**Figure 1:**
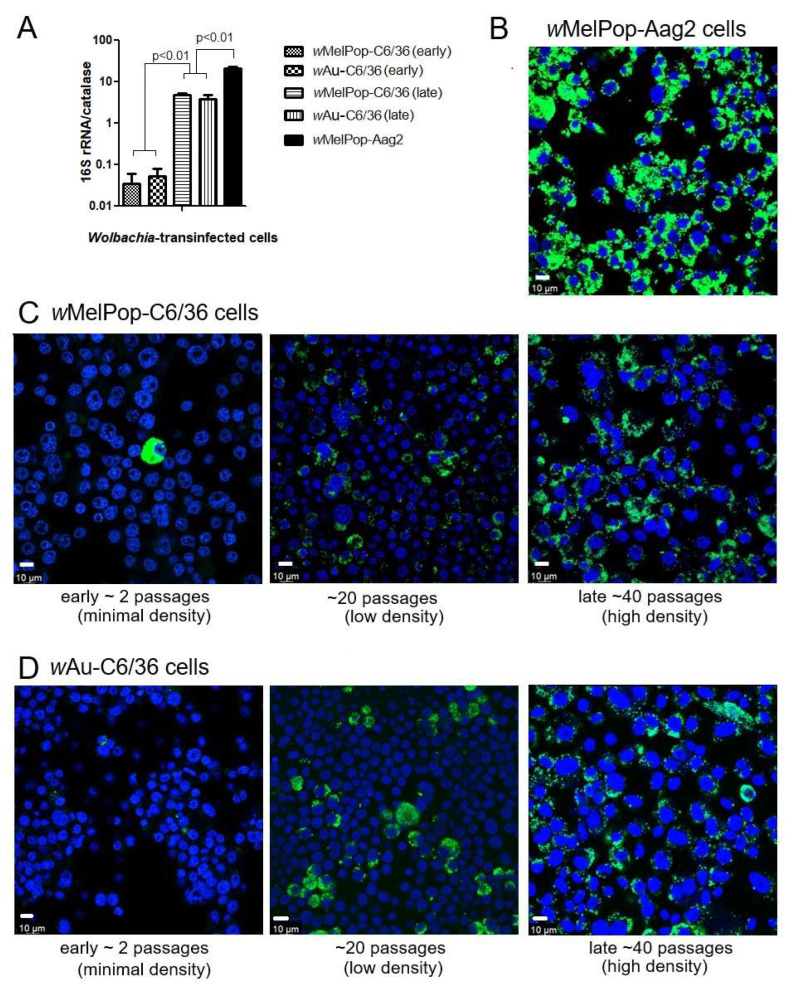
*Wolbachia* spread and density per cell. (**A**) qPCR for determining *Wolbachia* numbers/cell; *Wolbachia*’s “16S rRNA gene” numbers were compared with the cellular nuclear gene, “catalase”, gene numbers. DNA samples were extracted from cultured cells. qPCR determined the contained copy numbers of each gene by comparing against the standardized cDNA amount. (**B**) Fluorescent in situ hybridization (FISH). Anti-sense oligo DNA was probed to detect the 16S rRNA gene in *Wolbachia*. The anti-oligo DNA against the 16S r RNA gene, labeled by C3 fluorescence, were hybridized in *Wolbachia*-transinfected *w*MelPop-Aag2 cells. 16S rRNA was detected as highly intensified granules (Green fluorescence), compared with DAPI staining (Blue fluorescence). (**C**) FISH performed for *w*MelPop-C6/36 cells (early ~ late passage). (**D**) FISH performed for *w*AU-C6/36 cells (early ~ late passage).

**Figure 2:**
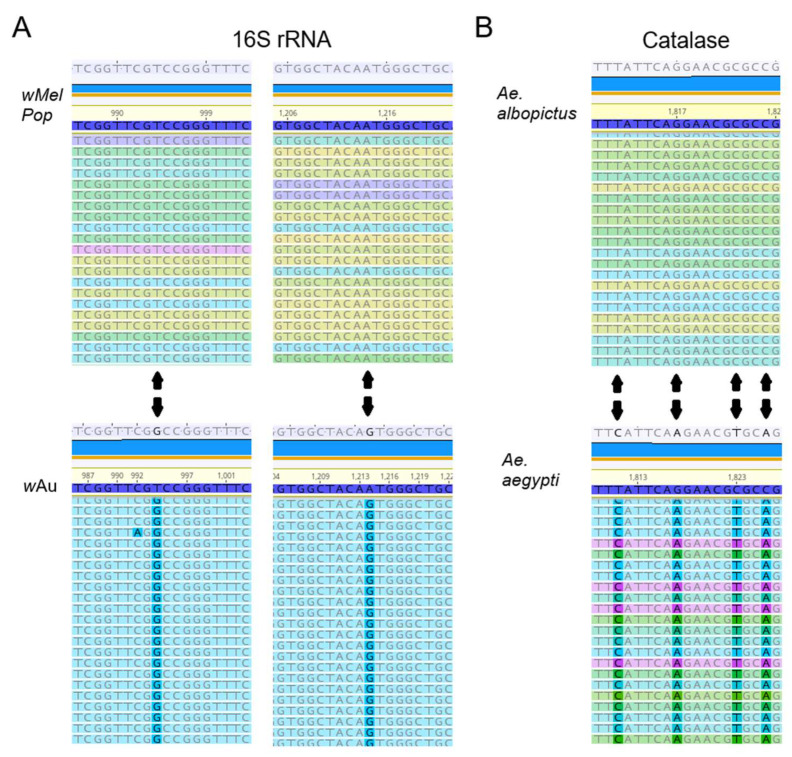
(**A**) Sequence differences of the 16S rRNA gene of *w*MelPop and *w*Au. RNA-seq data showed the two nucleotide differences of 16S rRNAs, matching the known *w*MelPop and *w*Au sequences (Supplemental Method File). (**B**) RNA-seq data of catalase transcript from samples matching the corresponding in *Ae. albopictus* and *Ae. aegypti*.

***w*MelPop or *w*Au spread through C6/36 cells and their densities increased via continuous cell culture.** We first quantified *Wolbachia* densities in the cells with qPCR. The *Wolbachia* gene 16S r RNA was targeted to compare the densities of *w*MelPop or *w*Au, while the “catalase” gene, which is localized at the mosquito nuclear genome, was used to determine cell numbers in the samples. When we started the cultures of these cells immediately after their arrival from WMP, the *w*MelPop density in Aag2 cells was the highest, ~20 copies/cell, in contrast to *w*MelPop and *w*Au in C6/36 cells, both of which contained the lowest *Wolbachia* density at ~1 copy/20 cells (Figure 1A). However, after the cell cultures were extended beyond 40 passages (the method is described in the Materials and Methods section), the densities of both *w*MelPop and *w*Au reached ~5 copies/cell (Figure 1A). These different *Wolbachia* densities were confirmed with fluorescent in situ hybridization (FISH) to detect 16S rRNA (Figure 1B). The FISH data were consistent with qPCR data; the fluorescent signals of *w*MelPop in Aag2 cells were the most intensified and spread all throughout the cells (Figure 1B), while those of *w*Au and *w*MelPop gradually increased after the cell passages increased (Figure 1C,D). We also confirmed the authenticity of *w*Au and *w*MelPop strains through the nucleotide differences in 16S rRNA, using RNA-seq (Figure 2).

**Figure 3:**
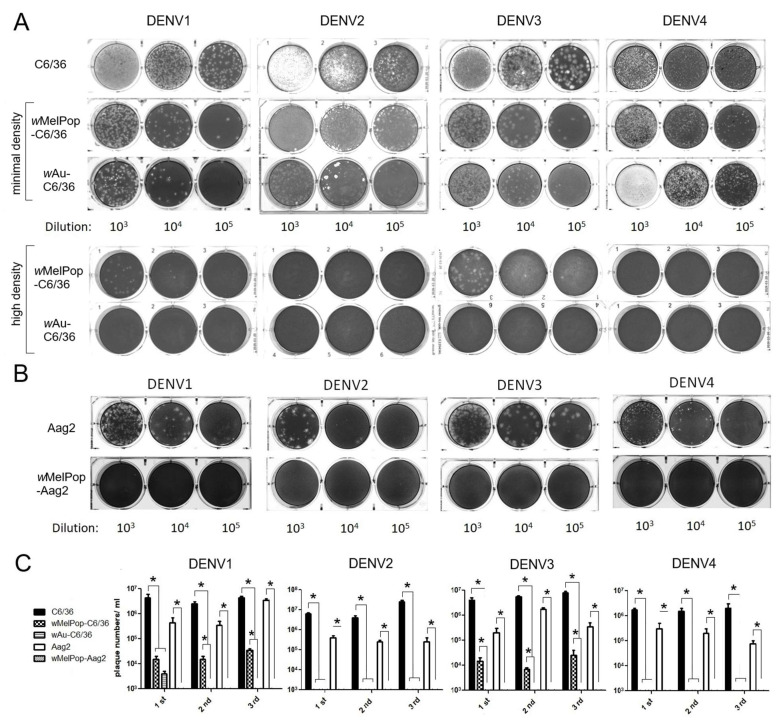
Plaque assay using LLC/MK2 cells. (**A**) DENV1-4 were used to infect the C6/36 mosquito cells (shown on the left in the pictures). After 5 days, the collected supernatants were diluted by 10^3^ to 10^5^ (shown as dilution at the bottom) to infect LLC/MK2 cells. Minimal or high density refers to the *Wolbachia* density in the infected C6/36 mosquito cells. (**B**) Aag2 cells were similarly infected and the collected supernatants were used to infect LLC/MK2 cells. (**C**) The experiment was repeated three different times. The plaque numbers were counted in each duplicated well. The numbers represent mean ± S.E. (n = 2) and are plotted using GraphPad Prism 5.0. * *p* < 0.01 was confirmed by one-way ANOVA with Bonferroni post-test.

**Infectious titers of all DENV1-4 serotypes were suppressed by the presence of *w*MelPop or *w*Au in C6/36 cells as well as Aag2 cells.** We examined the infectivity of DENV1-4 serotypes after replication in *Wolbachia*-containing mosquito cells. DENV1-4 serotypes were added at multiplicity of infection (MOI) 1 into cell cultures of *w*Au-C6/36 cells, *w*MelPop-C6/36 cells, or *w*MelPop-Aag2 cells, to compare with infecting naïve C6/36 cells and Aag2 cells. Supernatants from these cell cultures were collected at 5 days post-infection (p.i.) and tested for viral infectious titers by plaque assays in LLC/MK2 (monkey kidney) cells. All DENV serotypes showed significantly reduced or undetected plaque numbers after infecting *w*Au-C6/36 cells, *w*MelPop-C6/36 cells, or *w*MelPop-Aag2 cells, compared with DENV titers from naïve C6/36 cells (2–3 × 10^6^ PFU/mL) or those from Aag2 cells (1–9 × 10^5^ PFU/mL) (Figure 3).

**Figure 4:**
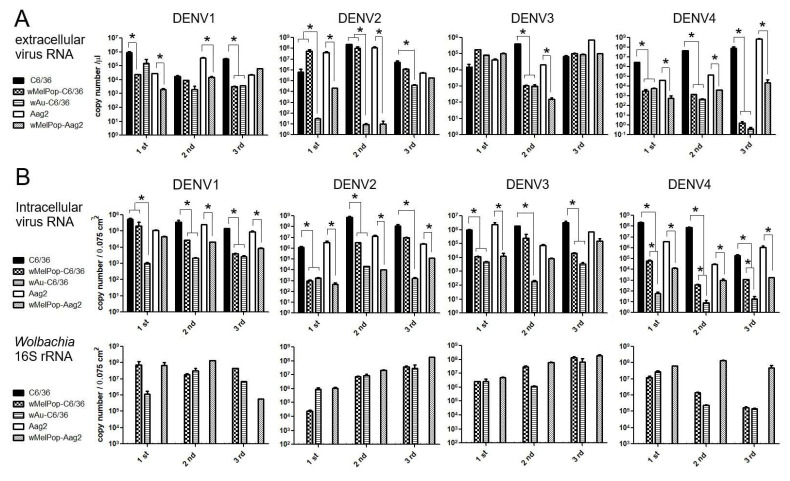
RT-qPCR for measuring DENV copy numbers. (**A**) Extracellular DENV copy numbers were calculated comparing the acquired Ct values with standard amounts of cDNA (see Materials and Methods). (**B**) Intracellular copy numbers of DENV (top) and 16S rRNA transcript (bottom). (See Materials and Methods). The viral RNA copy numbers represent mean ± S.E. (n = 2) and are plotted using GraphPad Prism 5.0. * *p* < 0.01 was confirmed by one-way ANOVA with Bonferroni post-test.

**Extracellular viral genome copy numbers were not correlated to the virus infectious titers.** DENV RNAs were extracted from the supernatants of the virus-infected cell cultures and were tested to determine the viral copy numbers by RT-qPCR. Either *w*Au- or *w*MelPop-containing C6/36 cells or Aag2 cells showed diverse effects on virus copy numbers in the supernatants among repeated experiments (Figure 4A). The presence of *Wolbachia* mostly reduced viral copies but occasionally had no effect. These discrepancies were not uniform among *Wolbachia* species, DENV serotypes, or mosquito cell types. Also, the reduced virus titers and the virus copy numbers for the *Wolbachia* were not all correlated (even the supernatants containing abundant virus copy numbers did not induce high virus titers). These results suggest that *Wolbachia* uniformly suppresses the infectivity of the produced DENV1-4 serotypes but variously affects the viral replication.

Intracellular viral copy numbers, corresponding to viral replication efficiencies, were also variably suppressed by *w*Au or *w*MelPop. We further tested if the intracellular virus copy numbers were correlated to the extracellular ones. At the time of collecting supernatants at 5 days p.i., we also extracted total RNAs from the cells. The intracellular virus copy numbers were compared with the amounts of *Wolbachia*’s 16S r RNA transcript (Figure 4B). The results indicated that the presence of either *w*Au *or w*MelPop mostly suppressed viral replication efficiency in both C6/36 cells and Aag2 cells, but the inhibitory effects on the reproduced virus numbers were not correlated with *Wolbachia* transcript amounts. Also, a discrepancy in the viral copy numbers between inside and outside cells was observed. The data suggested that the virus replication was not uniformly suppressed by *Wolbachia* density. It rather indicated that *Wolbachia* is involved in the suppression of various processes in the DENV life cycle.

***w*Au and *w*MelPop greatly altered C6/36 and Aag2 cellular gene expressions but did not affect pathway enrichment much.** The effects of *Wolbachia* in the cellular transcriptomes were examined by RNA-seq. Total RNAs were extracted from *w*Au- or *w*MelPop-C6/36 cells, *w*MelPop-Aag2 cells, and those naïve cells without *Wolbachia* and then were purified by ribosomal RNA depletion method. The fastq data were generated from RNA-seq reads, which then were mapped to the *Ae. albopictus* genome (GCF_035046485.1, Genebank) or *Ae. aegypti* genome (GCF_002204515.2, Genebank). The listed *Ae. albopictus* and *Ae. aegypti* genomes in the references contain 25,320 and 19,623 genes, respectively. The numbers of detected differential gene expressions from all C6/36 and Aag2 samples in our study ranged between the minimums of 15,380 and 14,134 and maximums of 22,954 and 18,117 transcripts, respectively (Supplemental File S1).

**Figure 5:**
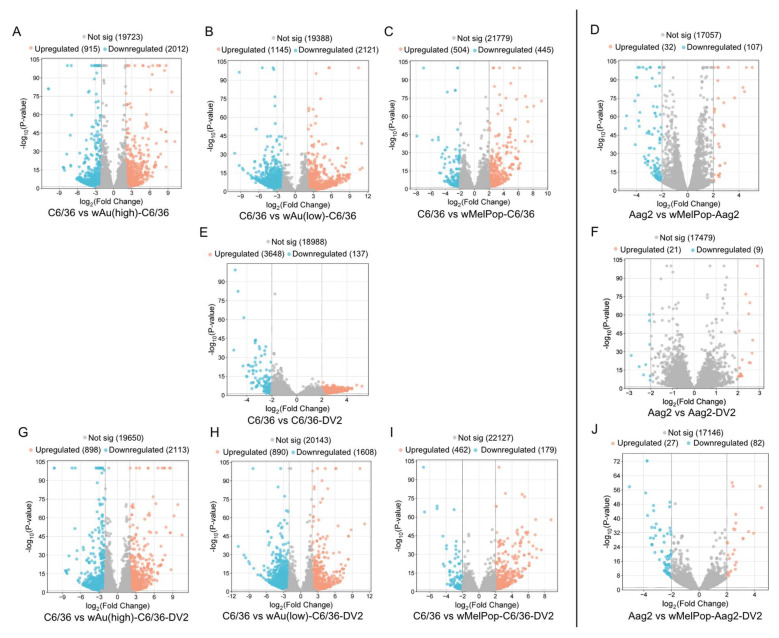
Volcano plots (global transcriptome changes) in response to *w*MelPop, *w*Au, DENV2 (DV2), or both infections in C6/36 cells (left) and Aag2 cells (right). Volcano plots visualize each gene’s Log_2_ Differential Expression Ratio (x-axis) and −log10 *p*-value (y-axis). Each plot represents an upregulated (orange), downregulated (blue), and non-significantly changed (gray) transcript. (**A**) *w*Au(high)-induced transcript in C6/36 cells (compared with naïve C6/36 cells). (**B**) *w*Au(low)-induced one. (**C**) *w*MelPop-induced one. (**D**) *w*MelPop-induced one in Aag2 cells (compared with naïve Aag2 cells). (**E**) DV2-induced one in C6/36 cells. (**F**) DV2-induced one in Aag2 cells. (**G**) DV2-induced one in *w*Au(high)-C6/36 (compared with naïve C6/36 cells). (**H**) DV2-induced one in *w*Au(low)-C6/36 cells. (**I**) DV2-induced one in *w*MelPop-C6/36 cells. (**J**) DV2-induced one in *w*MelPop-Aag2 (compared with naïve Aag2 cells).

**Figure 6:**
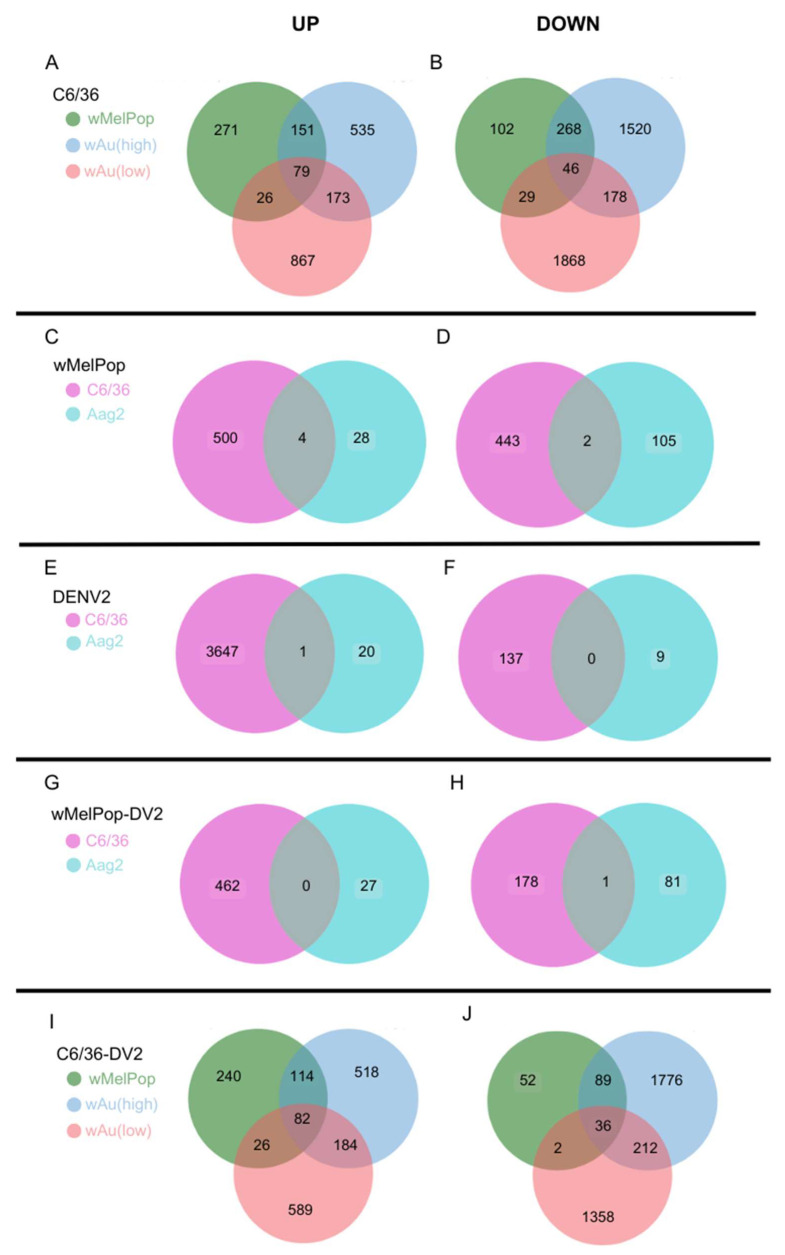
Venn diagrams showing overlapped, differentially expressed genes between conditions. Numbers of upregulated (Log_2_DER > 2, *p* < 0.05) or downregulated genes (Log_2_DER < −2, *p* < 0.05) were analyzed to determine the overlap among the different samples. (**A**,**B**) Upregulated (**A**) or downregulated (**B**) overlapped genes among *w*MelPop, high-density *w*Au, and low-density *w*Au-C6/36 cells. (**C**,**D**) Upregulated (**C**) or downregulated (**D**) overlapped genes in *w*MelPop-C6/36 and *w*MelPop-Aag2 cells. (**E**,**F**) Upregulated (**E**) or downregulated (**F**) overlapped genes in DENV2-infected C6/36 and DENV2-infected Aag2 cells. (**G**,**H**) Upregulated (**G**) and downregulated (**H**) overlapped genes in DENV2-infected *w*MelPop-C6/36 and DENV2-infected *w*MelPop-Aag2 cells. (**I**,**J**) Upregulated (**I**) and downregulated (**J**) overlapped genes among DENV2-infected *w*MelPop-C6/36 cells, DENV2-infected high-density *w*Au-C6/36 cells, and DENV2-infected low-density *w*Au-C6/36 cells.

*w*Au and *w*MelPop in C6/36 cells induced great alterations to the gene expression. As shown in Figure 5, high- or low-density *w*Au in C6/36 cells resulted in upregulation (Log_2_Differential Expression Ratio (DER) > 2, *p* < 0.05) of 915 or 1145 transcripts as well as in downregulation (Log_2_DER < −2, *p* < 0.05) of 2012 or 2121 transcripts, respectively (Figure 5A,B, Supplemental File S2). *w*MelPop in C6/36 cells also induced 504 upregulated and 445 downregulated transcripts (Figure 5C, Supplemental File S2). Among these different *Wolbachia* species- and density-containing C6/36 cells, 79 upregulated and 46 downregulated transcripts were commonly observed (Figure 6A,B, Supplemental File S3). Between high- and low-density *w*Au-C6/36 cells, 173 upregulated and 178 downregulated transcripts were shared. Similarly, *w*MelPop-C6/36 cells shared 151 upregulated and 268 downregulated transcripts with high-density *w*Au-C6/36 cells. In contrast, *w*MelPop-C6/36 cells had fewer shared transcripts with low-density *w*Au-C6/36 cells: 26 upregulated and 29 downregulated transcripts. Since the *Wolbachia* density of the examined *w*MelPop-C6/36 cells is close to that of high-density *w*Au-C6/36 cells (~5 copies/cell, Figure 1A), the same extent of density between different *Wolbachia* may similarly affect the transcriptome.

In contrast, Aag2 cells were less respondent to *w*MelPop; 32 transcripts were upregulated and 107 were downregulated (Figure 5D, Supplemental File S2). Between *w*MelPop-Aag2 cells and *w*MelPop-C6/36 cells, four upregulated and two downregulated transcripts were shared (Figure 6C,D, Supplemental File S3). These results may indicate that the *Wolbachia* effect on the cellular transcriptome is not uniform between mosquito species.

**Figure 7:**
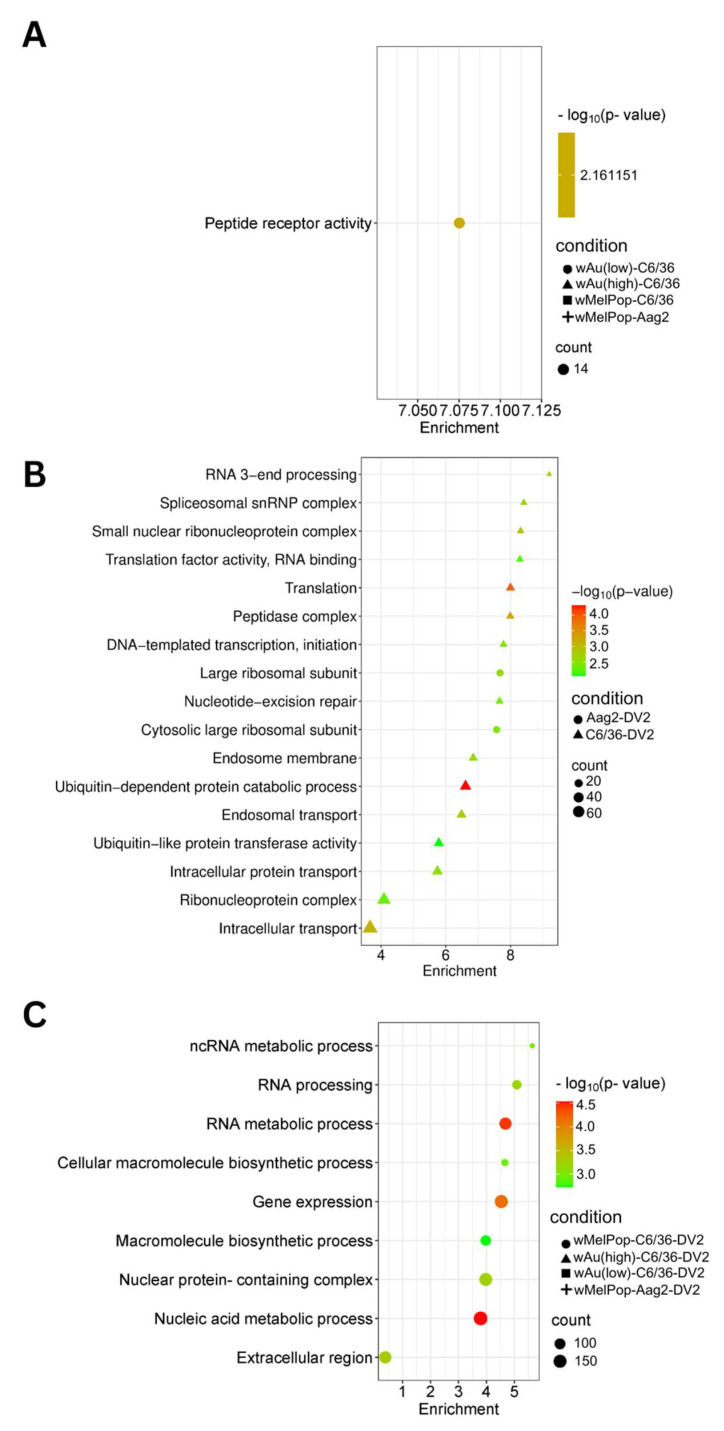
Pathways affected by upregulated genes in response to *w*MelPop or *w*Au transinfection, DENV2 infection, or both in C6/36 and Aag2 cells. GO Components, Functions, and Pathways were determined from overexpressed (Log_2_DER > 0) genes using StringDB. (**A**) Upregulated pathways identified in C6/36 cells and Aag2 cells containing *Wolbachia*. (**B**) Upregulated pathways identified in DENV2-infected C6/36 and Aag2 cells. (**C**) Upregulated pathways identified in DENV2-infected C6/36 cells and Aag2 containing *Wolbachia*. Pathways shown in this figure were the most relevant to the findings. Not all pathways are shown; refer to Supplemental File S4 for all pathways.

**Figure 8:**
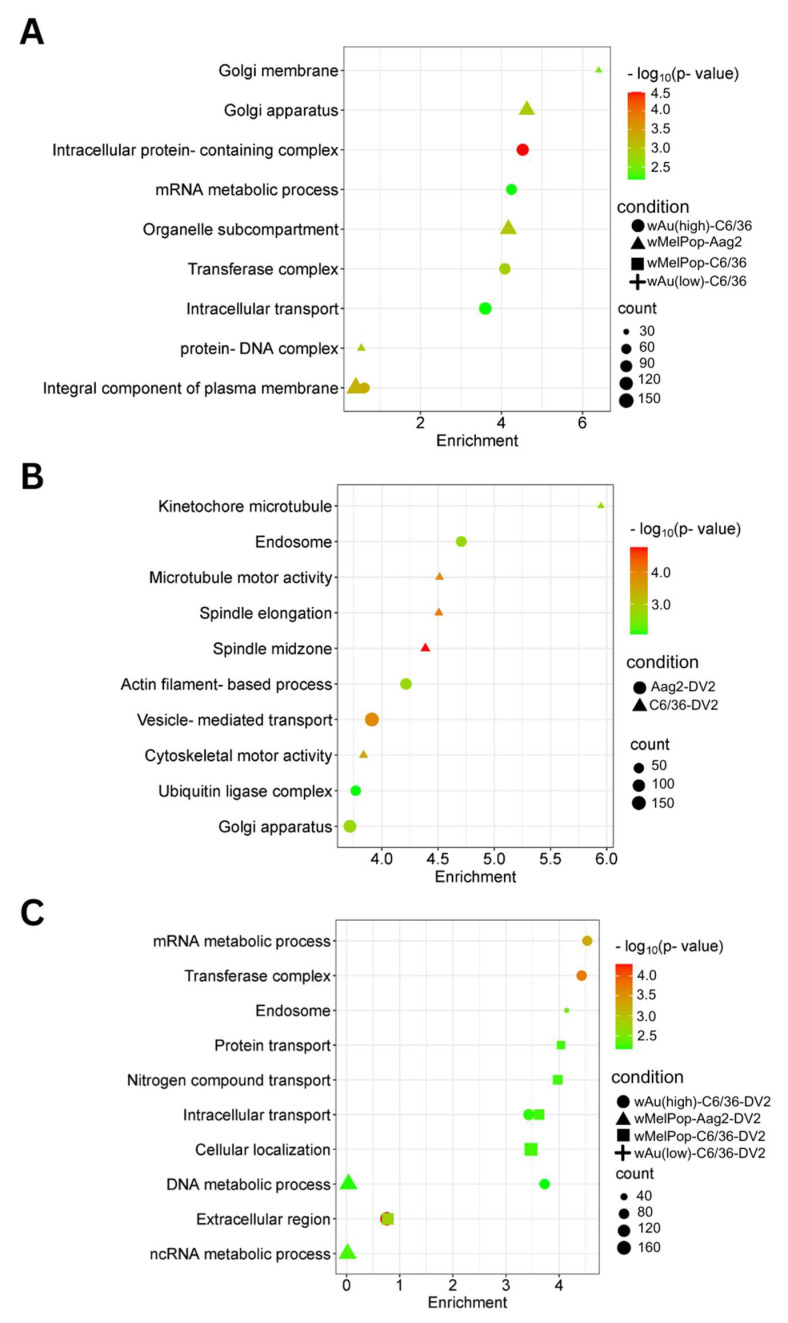
Pathways affected by downregulated genes in response to *w*MelPop or *w*Au transinfection, DENV2 infection, or both in C6/36 cells and Aag2 cells. GO Components, Functions, and Pathways were determined from underexpressed (Log_2_DER < 0) genes using StringDB. (**A**) Downregulated pathways in C6/36 cells and Aag2 cells containing *Wolbachia*. (**B**) Downregulated pathways identified in DENV2-infected C6/36 and Aag2 cells. (**C**) Downregulated pathways identified in DENV2-infected C6/36 and Aag2 cells containing *Wolbachia*. Pathways shown in this figure were the most relevant to the findings. Not all pathways are shown; refer to Supplemental File S4 for all pathways.

We categorized the global effects of *Wolbachia* on C6/36 cells by examining their enriched cellular pathways. We specifically analyzed Gene Ontology (GO) pathways and terms using StringDB. Significantly enriched pathways and keywords are shown in Figure 7 (upregulated genes) and Figure 8 (downregulated genes). Low-density *w*Au in C6/36 cells upregulated one pathway: peptide receptor activity (Figure 7A, Supplemental File S4). No upregulated gene pathways were detected in high-density *w*Au- or *w*MelPop-C6/36 cells (Figure 7A, Supplemental File S4). Also, no upregulated gene pathways were found in *w*MelPop-Aag2 cells. As for the downregulated gene pathways, high-density *w*Au-C6/36 cells affected 10 pathways, while no pathways were found in low-density *w*Au-C6/36 or *w*MelPop-C6/36 cells (Figure 8A, Supplemental File S4). In contrast, *w*MelPop-Aag2 cells induced 23 downregulated gene pathways. High-density *w*Au-C6/36 cells downregulated the mRNA metabolic process and intracellular transport pathways, while *w*MelPop-Aag2 cells suppressed Golgi membrane and Golgi apparatus functions (Figure 8A, Supplemental File S4). The data show that *w*Au and *w*MelPop may affect the cellular functions in RNA metabolism that are critical for viral RNA synthesis and affect the trans-Golgi network (TGN) that is imperative for viral particle transport, respectively. However, this effect also seems to be different among mosquito species.

*w*Au and *w*MelPop opposed DENV2-induced transcriptome alterations and pathways and suppressed transcripts and pathways necessary for the DENV life cycle in C6/36 cells. In naïve C6/36 cells, DENV2 significantly upregulated 3648 mRNA transcripts (Figure 5E). The presence of high- or low-density *w*Au or *w*MelPop blocked 3500 (95.9%), 3414 (93.6%), or 3544 (97.1%) transcript inductions, respectively (Supplemental File S5). Similarly, DENV2-downregulated transcripts in naïve C6/36 cells were blocked by these *Wolbachia*; among 137 genes downregulated by DENV2, high- or low-density *w*Au or *w*MelPop prevented downregulation of 121 (88.3%), 95 (69.3%), or 132 (96.3%) transcripts, respectively (Supplemental File S5).

In contrast, the numbers of the transcripts upregulated by DENV2 infection in the high- or low-density *w*Au-C6/36 cells or *w*MelPop-C6/36 cells were lower, as follows: 898, 890, or 462, respectively (Figure 5G,H,I, Supplemental File S2). Among all these *Wolbachia*-containing C6/36 cells, 82 transcripts overlapped (Figure 6I, Supplemental File S3). High- and low-density *w*Au-C6/36 cells under DENV2 infection shared 184 transcripts, while *w*MelPop-C6/36 shared 114 transcripts with high-density *w*Au-C6/36 cells and 26 transcripts with low-density *w*Au-C6/36 cells.

Downregulated transcripts in high- or low-density *w*Au- or *w*MelPop-C6/36 cells under DENV2 infection were abundantly observed; 2113, 1608, or 179, respectively (Figure 5G,H,I, Supplemental File S2). A total of 36 transcripts were common among all these cells (Figure 6J, Supplemental File S3). High- and low-density *w*Au-C6/36 cells shared 212 downregulated transcripts, while *w*MelPop-C6/36 shared 89 transcripts with high-density wAu-C6/36 cells and 2 transcripts with low-density *w*Au-C6/36 cells (Figure 6J, Supplemental File S3). Again, this data may relate to the similar densities between *w*MelPop and high-density-*w*Au in the C6/36 cells, as mentioned above.

GO Terms clarified the functions of these differentially expressed transcripts. A total of 120 GO Terms were affected by genes upregulated by DENV2 in naïve C6/36 cells, including many important homeostatic pathways and terms, such as the endosome membrane, ubiquitin-dependent protein metabolic process, endosomal transport, and intracellular transport (Figure 7B, Supplemental File S4). In contrast, the presence of *Wolbachia* induced different pathways, most of which relate to RNA metabolic processes (Figure 7C, Supplemental File S4). As for the downregulated gene pathways, DENV2 affected 50 GO Terms in C6/36 cells (Figure 8B, Supplemental File S4). However, these pathways were not affected in high-density *w*Au- or *w*MelPop-C6/36 cells (Supplemental File S4). Instead, high-density *w*Au affected 31 GO Terms, and *w*MelPop affected 6 GO Terms (Figure 8C, Supplemental File S4). High-density *w*Au- and *w*MelPop-C6/36 cells shared two downregulated gene pathways: protein transport and intracellular transport. No downregulated pathways were found in low-density *w*Au-C6/36 cells.

*w*MelPop also blocks induction of DENV2-upregulated transcripts and cellular pathways in Aag2 cells. Up- or downregulated transcripts under *w*MelPop were minimally correlated to pathway alterations. DENV2 infection altered fewer transcript distributions in Aag2 cells; only 21 or 9 transcripts were upregulated or downregulated, respectively (Figure 5F, Supplemental File S2). Only one upregulated gene was shared between DENV2-infected Aag2 cells and C6/36 cells: serine/threonine-protein phosphatase 6 regulatory ankyrin repeat subunit A isoform X7 (Figure 6E, Supplemental File S3). The function of the subunit is not well known. No downregulated transcripts were shared between these cells (Figure 6F, Supplemental File S3).

Two GO Terms were affected by upregulated genes in DENV2-infected Aag2 cells, both of which related to the large ribosomal subunit (Figure 7B, Supplemental File S4). A total of 38 GO Terms were affected by downregulated genes, including vesicle-mediated transport, ubiquitin ligase complex activity, and Golgi apparatus activity (Figure 8B, Supplemental File S4). Similar downregulated gene-caused GO Terms were shared between DENV2-infected C6/36 and Aag2 cells: ubiquitin-related pathways and intracellular transport.

*w*MelPop in Aag2 cells blocked the upregulation of 20 (95.2%) out of 21 transcripts and the downregulation of 7 (77.8%) out of 9 transcripts affected in response to DENV2 infection (Supplemental File S5). However, *w*MelPop-Aag2 cells upregulated 27 and downregulated 82 transcripts in response to DENV2 infection (Figure 5J, Supplemental File S2). In comparison to *w*MelPop-C6/36, infected by DENV2, no upregulated transcripts were shared, but 1 downregulated transcript was (Figure 6G,H, Supplemental File S3).

No upregulated gene pathways were found in *w*MelPop-Aag2 cells under DENV2 infection (Figure 7C, Supplemental File S4), but two downregulated gene pathways were observed: the DNA and ncRNA metabolic processes pathways (Figure 8C, Supplemental File S4). The DNA metabolic process pathway was also affected in high-density *w*Au-C6/36 cells under DENV2 infection.

**DENV genome alterations were rarely observed.** Previously, we reported DENV2 genome mutations after replicating in *w*MelPop-C6/36 cells, in which extracellular and intracellular viral replications were maintained, comparable to those in naïve C6/36 cells, despite the loss of infectivity to LLC/MK2 mammalian cells [48]. It was considered that the *w*MelPop suppressed wild-type DENV2 replication, leading to the selection of the mutated viral population which efficiently replicated in *w*MelPop-C6/36 cells but not in the mammalian cells. In this study, we examined if the same or similar mutations were induced in DENV genomes. RNA-seq analysis in the viral genomes did not show a newly appeared dominant population of DENV mutants after replicating in the Wolbachia-present mosquito cells, although there were minor quasispecies, mixed with low (<25%) appearance rates containing mutations (Supplemental Table File). The data suggested that *Wolbachia* causes an unfavorable environment for DENV replication. However, the lack of a new majority appearance indicates the varieties of *Wolbachia*’s suppressive mechanisms on DENV life cycles. One subtype which developed mutation(s) to overcome one suppressive process would encounter difficulties in utilizing the same mutation(s) in the subsequent stages of the viral life cycles, including packaging, trafficking, and maturation.

## 4. Discussion

It was reported that the density of *Wolbachia* in infected cells in culture could fluctuate by passaging; longer incubation (~7 days interval) together with higher cell confluence (~100%) is necessary to avoid the loss of *w*AlbB *Wolbachia* in *Ae. albopictus*-derived Aa23 cells [39]. In this study, we used *w*Au and *w*MelPop *Wolbachia*-transinfected C6/36 (*Ae. albopictus*) and Aag2 (*Ae. aegypti*) cells. *w*Au- or *w*MelPop-containing C6/36 cells initially had very low densities of *Wolbachia* (Figure 1). However, the extended culturing period between trypsin-used passaging, together with the high cell confluency, could increase *Wolbachia*-containing cell rates and their densities in C6/36 cells (Figure 1). In contrast, wMelPop-Aag2 cells contained diffusely spread high-density *Wolbachia* at the beginning of the cell culture. The differences in *Wolbachia* densities between C6/36 cells and Aag2 cells could be due to the initial cellular conditions provided by WMP, although the differences in cellular reactions against *Wolbachia* may also exist between these cells. *Wolbachia* gene 16S r RNA amounts were compared among samples through cell passages. As shown in Figure 4, there were variations of *Wolbachia*-derived 16S r RNA among different passaged cellular samples. It is considered that the cellular reactions to eradicate *Wolbachia* still exist after reaching high densities in cells. These reactions may be reflected in transcriptomic alterations, which could variously affect DENV replication and infectivity.

In general, DENV 4 serotypes have a large discrepancy in copy numbers compared to infectious titers in the supernatants of the infected cells [49,50,51]. RNA copy numbers exceed infectious titers by two to five orders of magnitude, which vary among flavivirus species, including DENV serotypes [51,52,53]. It is postulated that two processes in the DENV life cycle were involved: (1) the attachment; the very low rate (1 in 2600 to 1 in 72,000, depending on the assays used) of the produced virus particles capable of attaching to the cell surface; (2) the endocytosis and the fusion; only 1 in 6 attached virus particles were capable of fusing to cellular membranes [54]. The primary reason for this large discrepancy must be due to the former, the very inefficient virus attachment to the cells, while the latter, the limited virus entry by the endocytosis~fusion process, could further reduce the infectivity. Our data showed that the discrepancies in wild-type DENV1-4 were varied and larger in DENV2 and DENV4 than in DENV1 and DENV3. However, the presence of either *w*Au or *w*MelPop *Wolbachia* further increased the discrepancies (>100 times), suggesting that *Wolbachia* could affect the virus’s function of attaching to the cellular membrane. The mechanism has been also postulated by others (review in [55]).

DENV particles which are assembled at the ER are initially “immature”, but must be “matured” in order to attach to the cell surface [56]. This maturation relies on the conformational change of “E” protein in the surface of the DENV particle; the initial three E monomers per asymmetric unit must be changed to three parallel homodimers arranged in a herringbone pattern on the 30 rafts [57]. The lowered pH (around pH 6.0) in the peripheral trans-Golgi network (TGN) induces this E transformation, which enables the prM cleavage site to be accessible by the cellular furin protease [58,59]. After the prM cleavage, the remaining M protein stabilizes the herringbone E homodimers, completing DENV particle maturation [57,60]. The enlarged discrepancies between infectious titers and virus copy numbers are potentially due to an increased inefficiency in the maturation process. Furins recognize a minimum sequence of R-X-R/K/X-R (P4~P1) [61]. Therefore, one possibility was that the mutation(s) happened at the prM protein, including these recognition sequences. However, a “prM” mutation was not observed for the *Wolbachia* in this study or in the previous one [48]. It is rather considered that *Wolbachia* affects the E protein maturation process through TGN and furin activity.

DENV particle is made of the structural proteins C, prM, and E in the membrane, into which the synthesized RNA genome is packaged. Our data showed that extracellular viral copy numbers were suppressed in the *Wolbachia*-containing mosquito cells, suggesting that the processes from assembly/packaging to transportation were suppressed by the presence of *Wolbachia*. Several cellular factors play roles in flavivirus assembly in mammalian cells, including nucleolin, which binds RNA and subsequently interacts with the DENV C protein through the packaging [62]. DDX56, nucleolar RNA helicase which alters the secondary structure of RNA, also interacts with the flavivirus C protein, playing a role in packaging [63]. ALIX, which constructs the endosomal sorting complex required for transport (ESCRT), interacts with the DENV NS3 protein, through which ALIX expression affects extracellular DENV copy numbers [64]. TSG101, another subunit of the ESCRT-I complex (review in [65]), interacting with ATPase for membrane scission, an important step in vesicular transport and intracellular trafficking, is involved in flavivirus particle formation and the consequent budding process [66]. Mosquito genes corresponding to these mammalian proteins have not been clarified yet. However, our GO analysis based on transcriptome data indicated that intracellular transport and Golgi apparatus pathways were affected by *w*Au and/or *w*MelPop, clearly indicating that the lowered function of intracellular transport on TGN by *Wolbachia* could prevent the DENV transport.

The lowered intracellular virus amounts were also observed in some samples, which must be due to defects in viral replication efficiency. RNA-seq indicated the downregulation of RNA metabolism by *w*Au or *w*MelPop, suggesting the suppressing effect of *Wolbachia* on the cellular RNA machinery. However, it was also observed that the extracellular viral loads were not correlated to the lowered intracellular viral copy numbers. This phenomenon could be related to the suppression of the virus entry~fusion process, since the culturing period for 5 days after DENV infection is expected to infect DENV into regenerated cells multiple times. Therefore, the defect at the entry into cells could lower the intracellular virus amounts at 5 days p.i. DENV has been reported to attach to a variety of receptors: heparan sulfate, mannose receptor, laminin receptor, DC-SIGN, GRP78, and many unknown proteins (review in [67]). DENV entry via clathrin-mediated endocytosis was shown [68]. However, these molecules and mechanisms were analyzed in mammalian cells and the corresponding behaviors in mosquito cells remain unclear. Our RNA-seq results did not reveal the involvement of those molecules. In addition, the method we used, Trizol RNA extraction from cells, included intracellular replicating and packaged virus as well as the plasma membrane-bound virus. These viral RNAs in the different viral life cycles cannot be distinguished in this study. As a case for further study to distinguish the different stages’ viral RNAs, it is important to separate cellular plasma membrane-bound virus particles, intracellular replicating viral RNA, and packaged virus particles, in order to analyze their effects on the viral infection/replication mechanism.

It was observed that higher numbers of altered transcripts (Log_2_FC> 2 or <−2) are even correlated to lower numbers of GO enriched pathways and vice versa. *Wolbachia-*containing cells upregulated and downregulated numerous transcripts, although there were quite a few GO enriched pathways in these cells (Figure 7A and Figure 8A). In contrast, DENV2 infection altered many transcripts as well as GO pathways (Figure 7B and Figure 8B). It was revealed that *Wolbachia* presence in cells blocked these DENV-induced transcripts and GO pathways and induced different transcripts and GO pathways, all of which must play roles in antagonizing the processes necessary for the DENV life cycle. The data indicating that *Wolbachia* does not change the cellular function, pathway, and homeostasis means that *Wolbachia* lives with a symbiotic relationship in cells, different from virus infections, which drastically alter the cellular environments for themselves. Furthermore, *Wolbachia* could block the DENV-induced cellular environment and oppose the pathways necessary for DENV replication and infectivity.

Common up- or downregulated transcripts were observed among *Wolbachia-*infected C6/36 cells (Supplemental File S3). Some of the greatest upregulated and downregulated transcripts are listed in Table 1. Upregulated genes include (1) MD-2-related lipid-recognition protein, which is known to be a coreceptor for Toll-like receptor; (2) transcription factor SOX8, which functions throughout embryonic development; (3) teneurin-m isoform X6, which plays a role in pattern formation and morphogenesis; and (4) ubiquitin ligase (E3), which plays a crucial role in protein degradation in the recycling process. The first three transcripts remained at the highest induction under DENV2 infection (Table 2). Downregulated genes include (1) peroxidasin isoforms, which are involved in extracellular matrix formation; (2) mitochondrial translation initiation factor IF-2, which functions as the initiation of the protein synthesis within mitochondria; and (3) defense protein 1 (putative), which is known to play a role in the defense mechanism against pathogens in plant cells (Table 1). The former two transcripts remained highly downregulated under DENV2 infection (Table 2). The roles of all these cellular proteins in insects remained unclarified in terms of their effects on suppressing DENV life cycles. Further analyses are necessary to elucidate these key cellular proteins that antagonize DENV replication and infection. However, as we showed here, DENV replication and infectivity are suppressed at various levels through the cellular processes by *Wolbachia*, which prevents DENV-induced cellular pathways and instead induces different environments affecting the DENV life cycle.

## Figures and Tables

**Table 1:** The common transcripts altered among Wolbachia-containing C6/36 cells. All transcript values ≤ 0.05.

Upregulated				Downregulated			
Log_2_FC							
Transcript Name	*w*MelPop-C6/36	*w*Au(high)-C6/36	*w*Au(low)-C6/36	Transcript Name	*w*MelPop-C6/36	*w*Au(high)-C6/36	*w*Au(low)-C6/36
MD-2-related lipid-recognition protein	9.1625	9.6196	8.4275	peroxidasin isoform X1	−4.9233	−10.7727	−6.6590
teneurin-m isoform X6	6.0924	6.9800	7.3084	peroxidasin isoform X2	−4.9465	−10.7634	−6.6496
transcription factor Sox-8	5.2088	7.0671	4.9688	translation initiation factor IF-2, mitochondrial	−6.9719	−7.6391	−9.2186
E3 ubiquitin-protein ligase RNF19B isoform X1	2.0218	2.7460	3.2385	putative defense protein 1	−4.0223	−2.7899	−3.4146

**Table 2:** The common transcripts altered among Wolbachia-containing C6/36 cells, in response to DENV2. All transcripts’ *p* < 0.05.

Upregulated				Downregulated			
Log_2_FC							
Transcript Name	*w*MelPop-C6/36-DV2	*w*Au(high)-C6/36-DV2	*w*Au(low)-C6/36-DV2	Transcript Name	*w*MelPop-C6/36-DV2	*w*Au(high)-C6/36-DV2	*w*Au(low)-C6/36-DV2
MD-2-related lipid-recognition protein	8.7682	9.8485	8.7952	peroxidasin isoform X1	−3.95738	−10.3273	−5.7127
teneurin-m isoform X6	5.2464	7.1151	7.4804	peroxidasin isoform X2	−3.9594	−10.3179	−5.7031
protein tweety-2	7.0201	9.2355	10.3369	translation initiation factor IF-2, mitochondrial	−6.7522	−7.4237	−8.2949
transcription factor Sox-8	5.2453	7.0929	4.34077	chaoptin	−6.6285	−6.9461	−4.9129

## Data Availability

The RNA-seq data as raw reads are available as Fastq files in NCBI Short Read Archive (SRA) Data/Download Web page (The BioProject accession numbers, PRJNA1242886 and PRJNA1243338).

## Supplementary Materials

The following supporting information can be downloaded at: https://www.mdpi.com/article/10.3390/v17070922/s1, Supplemental File S1: All Transcripts, including the number of genes detected following the mapping of transcripts. Supplemental File S2: Log_2_ Differential Expression Ratios and *p*-values for all detected genes across all conditions. Supplemental File S3: Venn Diagram Genes (Overlapped transcripts). Supplemental File S4: Enriched GO terms for all conditions. Supplemental File S5: identification of genes no longer up- or downregulated following DENV2-infected cells containing *Wolbachia*. Supplemental Table File: primer sequences and viral mutations are listed. Supplemental Method File: primer design strategy and method are described.

## Abbreviations

DENV: dengue virus, ER: endoplasmic reticulum, TGN: trans-Golgi network, NS: non-structural protein, RT-qPCR: reverse transcription quantitative PCR, RNA-seq: Next Generation Sequence analysis.
