# Supplementary material for: Infections of Aedes Mosquito Cells by Wolbachia Strains wAu and wMelpop Modulate Host Cellular Transcriptomes Differently and Suppress Dengue Viral Replication
Source: Viruses. 2025 Jun 28;17(7):922. doi: 10.3390/v17070922 (PMC12299481; doi:10.3390/v17070922)
Supplement: Supplementary file 1 [file viruses-17-00922-s001.zip › supplemental method file to viruses 2025.pdf]

wMelPop 16s rRNA gene

Wolbachia endosymbiont of Drosophila melanogaster isolate wMelpop chro - Nucleotide - NCBI

16 S ribosomal RNA genomic DNA 1229741..1231245

```
aaatttgaga gtttgatcct
  1229761 agctcagaat gaacgctggc ggcaggccta acacatgcaa gtcgaacgga gttatattgt
  1229821 agcttgctat ggtataactt agtggcagac gggtgagtaa tgtataggaa tctacctagt
  1229881 agtacggaat aattgttgga aacggcaact aataccgtat acgccctacg ggggaaaaat
  1229941 ttattgctat tagatgagcc tatattagat tagctagttg gtggagtaat agcctaccaa
  1230001 ggcaatgatc tatagctgat ctgagaggat gatcagccac actggaactg agatacggtc
  1230061 cagactccta cgggaggcag cagtggggaa tattggacaa tgggcgaaag cctgatccag
  1230121 ccatgccgca tgagtgaaga aggcctttgg gttgtaaagc tcttttagtg aggaagataa
  1230181 tgacggtact cacagaagaa gtcctggcta actccgtgcc agcagccgcg gtaatacgga
  1230241 gagggctagc gttattcgga attattgggc gtaaagggcg cgtaggcgga ttagtaagtt
  1230301 aaaagtgaaa tcccaaggct caaccttgga attgctttta aaactgctaa tctagagatt
  1230361 gaaagaggat agaggaattc ctagtgtaga ggtgaaattc gtaaatatta ggaggaacac
  1230421 cagtggcgaa ggcgtctatc tggttcaaat ctgacgctga ggcgcgaagg cgtggggagc
  1230481 aaacaggatt agataccctg gtagtccacg ctgtaaacga tgaatgttaa atatgggaag
  1230541 ttttactttc tgtattacag ctaacgcgtt aaacattccg cctggggact acggtcgcaa
  1230601 gattaaaact caaaggaatt gacggggacc cgcacaagcg gtggagcatg tggtttaatt
  1230661 cgatgcaacg cgaaaaacct taccactcct tgacatggaa attataccta ttcgaaggga
  1230721 tagggtcggt tcgtccgggt ttcacacagg tgttgcatgg ctgtcgtcag ctcgtgtcgt
  1230781 gagatgttgg gttaagtccc gcaacgagcg caaccctcat ccttagttac catcaggtaa
  1230841 tgctggggac tttaaggaaa ctgccagtga taaactggag gaaggtgggg atgatgtcaa
  1230901 gtcatcatgg cccttatgga gtgggctaca cacgtgctac aatggtggct acaatgggct
  1230961 gcaaagtcgc gaggctaagc taatccctta aaagccatct cagttcggat tgtactctgc
  1231021 aactcgagtg catgaagttg gaatcgctag taatcgtgga tcagcacgcc acggtgaata
  1231081 cgttctcggg tcttgtacac actgcccgtc acgccatggg aattggtttc actcgaagct
  1231141 aacgacctaa ccgcaaggag ggagttattt aaagtgggat cggtgactgg ggtgaagtcg
  1231201 taacaaggta gcagtagggg aatctgcagc tggattacct cctta
```

wAu

Wolbachia endosymbiont of Drosophila simulans wAu genome assembly, chr - Nucleotide - NCBI

16S ribosomal RNA genome 1167907..1169353

```
              agag tttgatccta gctcagaatg aacgctggcg gcaggcctaa cacatgcaag
  1167961 tcgaacggag ttatattgta gcttgctatg gtataactta gtggcagacg ggtgagtaat
  1168021 gtataggaat ctacctagta gtacggaata attgttggaa acggcaacta ataccgtata
  1168081 cgccctacgg gggaaaaatt tattgctatt agatgagcct atattagatt agctagttgg
  1168141 tggagtaata gcctaccaag gcaatgatct atagctgatc tgagaggatg atcagccaca
  1168201 ctggaactga gatacggtcc agactcctac gggaggcagc agtggggaat attggacaat
  1168261 gggcgaaagc ctgatccagc catgccgcat gagtgaagaa ggcctttggg ttgtaaagct
  1168321 cttttagtga ggaagataat gacggtactc acagaagaag tcctggctaa ctccgtgcca
  1168381 gcagccgcgg taatacggag agggctagcg ttattcggaa ttattgggcg taaagggcgc
  1168441 gtaggcggat tagtaagtta aaagtgaaat cccaaggctc aaccttggaa ttgcttttaa
  1168501 aactgctaat ctagagattg aaagaggata gaggaattcc tagtgtagag gtgaaattcg
  1168561 taaatattag gaggaacacc agtggcgaag gcgtctatct ggttcaaatc tgacgctgag
  1168621 gcgcgaaggc gtggggagca aacaggatta gataccctgg tagtccacgc tgtaaacgat
  1168681 gaatgttaaa tatgggaagt tttactttct gtattacagc taacgcgtta aacattccgc
  1168741 ctggggacta cggtcgcaag attaaaactc aaaggaattg acggggaccc gcacaagcgg
  1168801 tggagcatgt ggtttaattc gatgcaacgc gaaaaacctt accactcctt gacatggaaa
  1168861 ttatacctat tcgaagggat agggtcggtt cggccgggtt tcacacaggt gttgcatggc
  1168921 tgtcgtcagc tcgtgtcgtg agatgttggg ttaagtcccg caacgagcgc aaccctcatc
  1168981 cttagttacc atcaggtaat gctggggact ttaaggaaac tgccagtgat aaactggagg
  1169041 aaggtgggga tgatgtcaag tcatcatggc ccttatggag tgggctacac acgtgctaca
  1169101 atggtggcta cagtgggctg caaagtcgcg aggctaagct aatcccttaa aagccatctc
  1169161 agttcggatt gtactctgca actcgagtgc atgaagttgg aatcgctagt aatcgtggat
  1169221 cagcacgcca cggtgaatac gttctcgggt cttgtacaca ctgcccgtca cgccatggga
  1169281 attggtttca ctcgaagcta acgacctaac cgcaaggagg gagttattta aagtgggatc
  1169341 ggtgactggg gtg
```

Alignment statistics for match #1 wMelPop (top) vs wAu (bottom)

Score	Expect	Identities	Gaps	Strand
2662 bits(1441)	0.0	1445/1447(99 %)	0/1447(0 %)	Plus/Plus

```
Query  8     AGAGTTTGATCCTAGCTCAGAATGAACGCTGGCGGCAGGCCTAACACATGCAAGTCGAAC  67
             ||||||||||||||||||||||||||||||||||||||||||||||||||||||||||||
Sbjct  1     AGAGTTTGATCCTAGCTCAGAATGAACGCTGGCGGCAGGCCTAACACATGCAAGTCGAAC  60

Query  68    GGAGTTATATTGTAGCTTGCTATGGTATAACTTAGTGGCAGACGGGTGAGTAATGTATAG  127
             ||||||||||||||||||||||||||||||||||||||||||||||||||||||||||||
Sbjct  61    GGAGTTATATTGTAGCTTGCTATGGTATAACTTAGTGGCAGACGGGTGAGTAATGTATAG  120
```

```
Query  128  GAATCTACCTAGTAGTACGGAATAATTGTTGGAAACGGCAACTAATACCGTATACGCCCT  187
            ||||||||||||||||||||||||||||||||||||||||||||||||||||||||||||
Sbjct  121  GAATCTACCTAGTAGTACGGAATAATTGTTGGAAACGGCAACTAATACCGTATACGCCCT  180

Query  188  ACGGGGGAAAAATTTATTGCTATTAGATGAGCCTATATTAGATTAGCTAGTTGGTGGAGT  247
            ||||||||||||||||||||||||||||||||||||||||||||||||||||||||||||
Sbjct  181  ACGGGGGAAAAATTTATTGCTATTAGATGAGCCTATATTAGATTAGCTAGTTGGTGGAGT  240

Query  248  AATAGCCTACCAAGGCAATGATCTATAGCTGATCTGAGAGGATGATCAGCCACACTGGAA  307
            ||||||||||||||||||||||||||||||||||||||||||||||||||||||||||||
Sbjct  241  AATAGCCTACCAAGGCAATGATCTATAGCTGATCTGAGAGGATGATCAGCCACACTGGAA  300

Query  308  CTGAGATACGGTCCAGACTCCTACGGGAGGCAGCAGTGGGGAATATTGGACAATGGGCGA  367
            ||||||||||||||||||||||||||||||||||||||||||||||||||||||||||||
Sbjct  301  CTGAGATACGGTCCAGACTCCTACGGGAGGCAGCAGTGGGGAATATTGGACAATGGGCGA  360

Query  368  AAGCCTGATCCAGCCATGCCGCATGAGTGAAGAAGGCCTTTGGGTTGTAAAGCTCTTTTA  427
            ||||||||||||||||||||||||||||||||||||||||||||||||||||||||||||
Sbjct  361  AAGCCTGATCCAGCCATGCCGCATGAGTGAAGAAGGCCTTTGGGTTGTAAAGCTCTTTTA  420

Query  428  GTGAGGAAGATAATGACGGTACTCACAGAAGAAGTCCTGGCTAACTCCGTGCCAGCAGCC  487
            ||||||||||||||||||||||||||||||||||||||||||||||||||||||||||||
Sbjct  421  GTGAGGAAGATAATGACGGTACTCACAGAAGAAGTCCTGGCTAACTCCGTGCCAGCAGCC  480

Query  488  GCGGTAATACGGAGAGGGCTAGCGTTATTCGGAATTATTGGGCGTAAAGGGCGCGTAGGC  547
            ||||||||||||||||||||||||||||||||||||||||||||||||||||||||||||
Sbjct  481  GCGGTAATACGGAGAGGGCTAGCGTTATTCGGAATTATTGGGCGTAAAGGGCGCGTAGGC  540

Query  548  GGATTAGTAAGTTAAAAGTGAAATCCCAAGGCTCAACCTTGGAATTGCTTTTAAAACTGC  607
            ||||||||||||||||||||||||||||||||||||||||||||||||||||||||||||
Sbjct  541  GGATTAGTAAGTTAAAAGTGAAATCCCAAGGCTCAACCTTGGAATTGCTTTTAAAACTGC  600

Query  608  TAATCTAGAGATTGAAAGAGGATAGAGGAATTCCTAGTGTAGAGGTGAAATTCGTAAATA  667
            ||||||||||||||||||||||||||||||||||||||||||||||||||||||||||||
Sbjct  601  TAATCTAGAGATTGAAAGAGGATAGAGGAATTCCTAGTGTAGAGGTGAAATTCGTAAATA  660

Query  668  TTAGGAGGAACACCAGTGGCGAAGGCGTCTATCTGGTTCAAATCTGACGCTGAGGCGCGA  727
            ||||||||||||||||||||||||||||||||||||||||||||||||||||||||||||
Sbjct  661  TTAGGAGGAACACCAGTGGCGAAGGCGTCTATCTGGTTCAAATCTGACGCTGAGGCGCGA  720
```

```
Query  728   AGGCGTGGGGAGCAAACAGGATTAGATACCCTGGTAGTCCACGCTGTAAACGATGAATGT  787
             ||||||||||||||||||||||||||||||||||||||||||||||||||||||||||||
Sbjct  721   AGGCGTGGGGAGCAAACAGGATTAGATACCCTGGTAGTCCACGCTGTAAACGATGAATGT  780

Query  788   TAAATATGGGAAGTTTTACTTTCTGTATTACAGCTAACGCGTTAAACATTCCGCCTGGGG  847
             ||||||||||||||||||||||||||||||||||||||||||||||||||||||||||||
Sbjct  781   TAAATATGGGAAGTTTTACTTTCTGTATTACAGCTAACGCGTTAAACATTCCGCCTGGGG  840

Query  848   ACTACGGTCGCAAGATTAAAACTCAAAGGAATTGACGGGGACCCGCACAAGCGGTGGAGC  907
             ||||||||||||||||||||||||||||||||||||||||||||||||||||||||||||
Sbjct  841   ACTACGGTCGCAAGATTAAAACTCAAAGGAATTGACGGGGACCCGCACAAGCGGTGGAGC  900

Query  908   ATGTGGTTTAATTCGATGCAACGCGAAAAACCTTACCACTCCTTGACATGGAAATTATAC  967
             ||||||||||||||||||||||||||||||||||||||||||||||||||||||||||||
Sbjct  901   ATGTGGTTTAATTCGATGCAACGCGAAAAACCTTACCACTCCTTGACATGGAAATTATAC  960

Query  968   CTATTCGAAGGGATAGGGTCGGTTCGTCCGGGTTTCACACAGGTGTTGCATGGCTGTCGT  1027
             |||||||||||||||||||||||||| |||||||||||||||||||||||||||||||||
Sbjct  961   CTATTCGAAGGGATAGGGTCGGTTCGGCCGGGTTTCACACAGGTGTTGCATGGCTGTCGT  1020

Query  1028  CAGCTCGTGTCGTGAGATGTTGGGTTAAGTCCCGCAACGAGCGCAACCCTCATCCTTAGT  1087
             ||||||||||||||||||||||||||||||||||||||||||||||||||||||||||||
Sbjct  1021  CAGCTCGTGTCGTGAGATGTTGGGTTAAGTCCCGCAACGAGCGCAACCCTCATCCTTAGT  1080

Query  1088  TACCATCAGGTAATGCTGGGGACTTTAAGGAAACTGCCAGTGATAAACTGGAGGAAGGTG  1147
             ||||||||||||||||||||||||||||||||||||||||||||||||||||||||||||
Sbjct  1081  TACCATCAGGTAATGCTGGGGACTTTAAGGAAACTGCCAGTGATAAACTGGAGGAAGGTG  1140

Query  1148  GGGATGATGTCAAGTCATCATGGCCCTTATGGAGTGGGCTACACACGTGCTACAATGGTG  1207
             ||||||||||||||||||||||||||||||||||||||||||||||||||||||||||||
Sbjct  1141  GGGATGATGTCAAGTCATCATGGCCCTTATGGAGTGGGCTACACACGTGCTACAATGGTG  1200

Query  1208  GCTACAATGGGCTGCAAAGTCGCGAGGCTAAGCTAATCCCTTAAAAGCCATCTCAGTTCG  1267
             |||||| |||||||||||||||||||||||||||||||||||||||||||||||||||||
Sbjct  1201  GCTACAGTGGGCTGCAAAGTCGCGAGGCTAAGCTAATCCCTTAAAAGCCATCTCAGTTCG  1260

Query  1268  GATTGTACTCTGCAACTCGAGTGCATGAAGTTGGAATCGCTAGTAATCGTGGATCAGCAC  1327
             ||||||||||||||||||||||||||||||||||||||||||||||||||||||||||||
Sbjct  1261  GATTGTACTCTGCAACTCGAGTGCATGAAGTTGGAATCGCTAGTAATCGTGGATCAGCAC  1320
```

```
Query  1328  GCCACGGTGAATACGTTCTCGGGTCTTGTACACACTGCCCGTCACGCCATGGGAATTGGT  1387
             ||||||||||||||||||||||||||||||||||||||||||||||||||||||||||||
Sbjct  1321  GCCACGGTGAATACGTTCTCGGGTCTTGTACACACTGCCCGTCACGCCATGGGAATTGGT  1380

Query  1388  TTCACTCGAAGCTAACGACCTAACCGCAAGGAGGGAGTTATTTAAAGTGGGATCGGTGAC  1447
             ||||||||||||||||||||||||||||||||||||||||||||||||||||||||||||
Sbjct  1381  TTCACTCGAAGCTAACGACCTAACCGCAAGGAGGGAGTTATTTAAAGTGGGATCGGTGAC  1440

Query  1448  TGGGGTG  1454
             |||||||
Sbjct  1441  TGGGGTG  1447
```

Mosquito catalase gene

Aedes albopictus:

LOC109430890 catalase [Aedes albopictus (Asian tiger mosquito)] - Gene - NCBI

Aedes albopictus strain Foshan chromosome 2, AalbF5, whole genome shot - Nucleotide - NCBI

```
Gene 1..37411
                     /gene="LOC109430890"
                     /note="catalase; Derived by automated computational
                     analysis using gene prediction method: Gnomon."
                     /db_xref="GeneID:109430890"
     mRNA            join(1..444,35675..37411)
                     /gene="LOC109430890"
                     /product="catalase, transcript variant X1"
                     /experiment="COORDINATES: polyA evidence [ECO:0006239]"
                     /transcript_id="XM_019706975.3"
                     /db_xref="GeneID:109430890"
     mRNA            join(4..444,26463..28072)
                     /gene="LOC109430890"
                     /product="catalase, transcript variant X2"
                     /experiment="COORDINATES: polyA evidence [ECO:0006239]"
                     /transcript_id="XM_019706976.3"
                     /db_xref="GeneID:109430890"
     CDS             join(391..444,35675..37138) X1
                     join(391..444,26463..27923) x2

focus on the variant X1 ex2  CDS 35675..37138

gacaaa tccgtggcaa ctaccggcaa
    35701 tggtgctcca ttgggtacca aaacggccac tgctaccgtt ggcgaacggg gccctgtggt
    35761 gctgcaggat gtccacttcc tcgacgagat gtcccacttc gaccgtgagc gtattccgga
    35821 acgtgttgtg cacgccaagg gtgcaggtgc tttcggatac ttcgaggtaa ctcacgatat
    35881 cacccagtac tgtgcggcca aagtgttcga aaaggtcggc aagaaaactc cacttgccgt
    35941 gcgtttctcc actgttggtg gtgaaagcgg atcggccgat accgctcgtg atccacgtgg
    36001 tttcgccgta aaattctaca ccgatgatgg tgtgtgggat ttggtcggaa acaacactcc
    36061 gatcttcttc atccgcgatc caattctgtt ccccagcttc atccacaccc agaaacgtaa
    36121 cccagctact cacctgaagg atgccgatat gttctgggac ttcatctccc tgcgtccgga
    36181 aaccacccat caagtgatgt tcttgttcgc tgatcgtggt attcctgatg gctaccggtt
    36241 catgaacggc tatggatccc acaccttcaa gcttatcaat gctgatggaa agccggtgta
    36301 ctgtaagttc cacttcaaat gtaatcaagg tatcaaaaac ttggaagcca gacgagctga
    36361 tgaacttgct ggatctgatc ctgactatag cattcgggat ctgtacaacg caatcgccaa
    36421 gggtgagtac ccaagctgga atttgaaaat ccaggttatg acattcgaac aagctgagca
    36481 acatagcttc aacccgttcg atgtaactaa ggtctggcca cagaatgagt tcccattgat
    36541 tccggtcggt cgtatggtgc ttgatcgcaa tccaagcaac tactttgctg aggttgagca
    36601 aatcgctttt gctccatcgc acctggttcc aggtatcgag gcatccccgg acaagatgtt
    36661 gcaaggtcgt ctcttctctt acgctgatac gcaccgccat cgcctcggag caaactatct
    36721 tcaactgccg gtcaactgcc cgtaccgcgt tgccatgaag aactatcagc gtgatggccc
    36781 gatgaatgtc accgacaatc aaggaggagc cccgaactat tatcccaact ccttcgcagg
    36841 accggaaccg tgcggatttg caaataagct ccaaaactcc aaattccacg tgtccggaga
    36901 tgtcaaccgg ttcgagtccg gcgataccga ggacaatttc gcccagcccg ccattttcta
    36961 ccgccgcgtg ctggatgaag ccgcccgaga gcgtatgatc accaacatgg tcaaccacat
    37021 gtcggccgcc tcgtccttta ttcaggaacg cgccgtcaag aatttctcgc aagtcgatgc
    37081 cgattttgga cgtcgcttga ccgagggtct gaagctacgc cgttcggcca aaatgtaa
```

Aedes aegypti:

LOC5577893 catalase [Aedes aegypti (yellow fever mosquito)] - Gene - NCBI

Aedes aegypti strain LVP_AGWG chromosome 2, AaegL5.0 Primary Assembly, - Nucleotide - NCBI

```
Gene 1..50432
                     /gene="LOC5577893"
                     /note="Derived by automated computational analysis using
                     gene prediction method: Gnomon."
                     /db_xref="GeneID:5577893"
     mRNA            join(1..407,48705..50432)
                     /gene="LOC5577893"
                     /product="catalase, transcript variant X1"
                     /note="Derived by automated computational analysis using
                     gene prediction method: Gnomon. Supporting evidence
                     includes similarity to: 1 mRNA, 28 ESTs, 10 long SRA
                     reads, 50 Proteins, and 100% coverage of the annotated
                     genomic feature by RNAseq alignments, including 106
                     samples with support for all annotated introns"
                     /transcript_id="XM_001663550.3"
                     /db_xref="GeneID:5577893"
     mRNA            join(4..407,31938..33573)
                     /gene="LOC5577893"
                     /product="catalase, transcript variant X2"
                     /note="Derived by automated computational analysis using
                     gene prediction method: Gnomon. Supporting evidence
                     includes similarity to: 2 mRNAs, 52 ESTs, 8 long SRA
                     reads, 47 Proteins, and 100% coverage of the annotated
                     genomic feature by RNAseq alignments, including 112
                     samples with support for all annotated introns"
                     /transcript_id="XM_011495417.2"
                     /db_xref="GeneID:5577893"
     CDS             join(354..407,48705..50168) X1
                     join(354..407,31938..33398) X2
```

focus on the variant X1 ex2 CDS 48705..50168

```
gacaaa tccgttgcca
    48721 ctaccggcaa tggtgctcca ttgggtacta aaacggccac cgcaaccgtt ggtgagcgtg
    48781 gaccagttgt gctacaggat gtccatttcc tcgatgagat gtcccacttt gaccgggagc
    48841 gcattccgga acgagttgtg cacgccaagg gcgcaggtgc attcggatac ttcgaggtaa
    48901 cgcacgatat cacccagtat tgtgcggcca aagttttcga aaaggtcggc aagaaaaccc
    48961 cgctcgctgt tcgattctcc acggtgggcg gtgaaagtgg atcagccgat accgctcgtg
    49021 atccacgtgg tttcgctgtg aaattctaca ccgacgacgg tgtgtgggat ttggtcggaa
    49081 ataacactcc gatcttcttc attcgcgatc ccatcctgtt cccaagcttc atccataccc
    49141 agaagcgtaa cccagctact cacctgaagg atgccgatat gttctgggat ttcatctctc
    49201 tgcgaccaga atctacccat caagtgatgt tcctgttcgc tgaccgtggt attcccgatg
    49261 gctatcgatt catgaacggc tacggatcac atacgttcaa gcttatcaat gctcaaggaa
    49321 aaccagtgta ctgtaagttc cacttcaaat caaaccaagg catcaaaaac ctggaggcaa
    49381 gacgagctga cgaacttgct ggatctgatc ctgactatag cattcgcgat ctgtacaacg
    49441 ccatcgccaa aggagagtgc ccgagctgga atttgaagat tcaggtcatg accttcgaac
    49501 aagctgaaca acatagcttc aatccattcg atgtgactaa agtttggcca caaaatgagt
    49561 tccctctgat tccagttggc cgcatggtgc tggatcgcaa tccaagcaac tactttgctg
```

```
49621 aggttgagca aatcgctttt gctccttcgc atttggttcc tggtatcgag gcatccccgg
49681 acaagatgtt gcaaggtcgt ctcttctcct acgctgacac tcaccgtcat cgtctgggag
49741 caaactatct tcaactgccg gttaactgtc cgtatcgcgt ttctatgaaa aactaccaac
49801 gtgacggccc gatgaatgtt accgacaatc aaggtggcgc cccgaactac tatcccaatt
49861 ccttcggtgg accagaacca tgtggatttg cgcataagct tcaaaattcc aaattcaatg
49921 tttctggaga tgtaaaccgt ttcgagtccg gtgaaaccga agacaacttc gcccaacccg
49981 gcattttcta tcgccgtgtg ttggatgaag ctgctcgtga gcgcatgatc accaacatgg
50041 ttaatcacat gtccgcagct tcgcccttca ttcaagaacg tgcagttcaa aatttctcgc
50101 aagttgatgc tgactttgga cgccgtttga ccgagggctt gaaactgcgc cgctcagcca
50161 aaatgtaa
```

Range 1: 1 to 1464GraphicsNext MatchPrevious Match

Alignment statistics for match #1 9, albopictus (top) versus aegypti(bottom)

Score	Expect	Identities	Gaps	Strand
1757 bits(951)	0.0	1294/1465(88%)	2/1465(0%)	Plus/Plus

```
Query  1    GACAAATCCGTGGCAACTACCGGCAATGGTGCTCCATTGGGTACCAAAACGGCCACTGCT  60
            ||||||||||| || ||||||||||||||||||||||||||||| ||||||||||| ||
Sbjct  1    GACAAATCCGTTGCCACTACCGGCAATGGTGCTCCATTGGGTACTAAAACGGCCACCGCA  60

Query  61   ACCGTTGGCGAACGGGGCCCTGTGGTGCTGCAGGATGTCCACTTCCTCGACGAGATGTCC  120
            |||||||| || || || || || ||||| ||||||||||| |||||||| |||||||||
Sbjct  61   ACCGTTGGTGAGCGTGGACCAGTTGTGCTACAGGATGTCCATTTCCTCGATGAGATGTCC  120

Query  121  CACTTCGACCGTGAGCGTATTCCGGAACGTGTTGTGCACGCCAAGGGTGCAGGTGCTTTC  180
            ||||| ||||| ||||| ||||||||||| ||||||||||||||||| |||||||| |||
Sbjct  121  CACTTTGACCGGGAGCGCATTCCGGAACGAGTTGTGCACGCCAAGGGCGCAGGTGCATTC  180

Query  181  GGATACTTCGAGGTAACTCACGATATCACCCAGTACTGTGCGGCCAAAGTGTTCGAAAAG  240
            ||||||||||||||||| ||||||||||||||||| |||||||||||||| |||||||||
Sbjct  181  GGATACTTCGAGGTAACGCACGATATCACCCAGTATTGTGCGGCCAAAGTTTTCGAAAAG  240

Query  241  GTCGGCAAGAAAACTCCACTTGCCGTGCGTTTCTCCACTGTTGGTGGTGAAAGCGGATCG  300
            |||||||||||||| || || || || || |||||||| || || |||||||| |||||
Sbjct  241  GTCGGCAAGAAAACCCCGCTCGCTGTTCGATTCTCCACGGTGGGCGGTGAAAGTGGATCA  300

Query  301  GCCGATACCGCTCGTGATCCACGTGGTTTCGCCGTAAAATTCTACACCGATGATGGTGTG  360
            |||||||||||||||||||||||||||||||| || |||||||||||||| || ||||||
Sbjct  301  GCCGATACCGCTCGTGATCCACGTGGTTTCGCTGTGAAATTCTACACCGACGACGGTGTG  360

Query  361  TGGGATTTGGTCGGAAACAACACTCCGATCTTCTTCATCCGCGATCCAATTCTGTTCCCC  420
            ||||||||||||||||| |||||||||||||||||||| |||||||| || ||||||||
Sbjct  361  TGGGATTTGGTCGGAAATAACACTCCGATCTTCTTCATTCGCGATCCCATCCTGTTCCCA  420
```

```
Query  421   AGCTTCATCCACACCCAGAAACGTAACCCAGCTACTCACCTGAAGGATGCCGATATGTTC  480
             ||||||||||| |||||||| |||||||||||||||||||||||||||||||||||||||
Sbjct  421   AGCTTCATCCATACCCAGAAGCGTAACCCAGCTACTCACCTGAAGGATGCCGATATGTTC  480

Query  481   TGGGACTTCATCTCCCTGCGTCCGGAAACCACCCATCAAGTGATGTTCTTGTTCGCTGAT  540
             ||||| |||||||| ||||| || ||| | |||||||||||||||||| ||||||||||
Sbjct  481   TGGGATTTCATCTCTCTGCGACCAGAATCTACCCATCAAGTGATGTTCCTGTTCGCTGAC  540

Query  541   CGTGGTATTCCTGATGGCTACCGGTTCATGAACGGCTATGGATCCCACACCTTCAAGCTT  600
             ||||||||||| |||||||| || |||||||||||||| ||||| || || |||||||||
Sbjct  541   CGTGGTATTCCCGATGGCTATCGATTCATGAACGGCTACGGATCACATACGTTCAAGCTT  600

Query  601   ATCAATGCTGATGGAAAGCCGGTGTACTGTAAGTTCCACTTCAAATGTAATCAAGGTATC  660
             ||||||||| | ||||| || |||||||||||||||||||||||||  || ||||| |||
Sbjct  601   ATCAATGCTCAAGGAAAACCAGTGTACTGTAAGTTCCACTTCAAATCAAACCAAGGCATC  660

Query  661   AAAAACTTGGAAGCCAGACGAGCTGATGAACTTGCTGGATCTGATCCTGACTATAGCATT  720
             |||||| |||| || ||||||||||| |||||||||||||||||||||||||||||||||
Sbjct  661   AAAAACCTGGAGGCAAGACGAGCTGACGAACTTGCTGGATCTGATCCTGACTATAGCATT  720

Query  721   CGGGATCTGTACAACGCAATCGCCAAGGGTGAGTACCCAAGCTGGAATTTGAAAATCCAG  780
             || |||||||||||||| |||||||| || |||| ||| |||||||||||||| || |||
Sbjct  721   CGCGATCTGTACAACGCCATCGCCAAAGGAGAGTGCCCGAGCTGGAATTTGAAGATTCAG  780

Query  781   GTTATGACATTCGAACAAGCTGAGCAACATAGCTTCAACCCGTTCGATGTAACTAAGGTC  840
             || ||||| |||||||||||||| |||||||||||||| || |||||||| ||||| ||
Sbjct  781   GTCATGACCTTCGAACAAGCTGAACAACATAGCTTCAATCCATTCGATGTGACTAAAGTT  840

Query  841   TGGCCACAGAATGAGTTCCCATTGATTCCGGTCGGTCGTATGGTGCTTGATCGCAATCCA  900
             |||||||| |||||||||||  ||||||| || || || |||||||| ||||||||||||
Sbjct  841   TGGCCACAAAATGAGTTCCCTCTGATTCCAGTTGGCCGCATGGTGCTGGATCGCAATCCA  900

Query  901   AGCAACTACTTTGCTGAGGTTGAGCAAATCGCTTTTGCTCCATCGCACCTGGTTCCAGGT  960
             ||||||||||||||||||||||||||||||||||||||||| |||||  ||||||| |||
Sbjct  901   AGCAACTACTTTGCTGAGGTTGAGCAAATCGCTTTTGCTCCTTCGCATTTGGTTCCTGGT  960

Query  961   ATCGAGGCATCCCCGGACAAGATGTTGCAAGGTCGTCTCTTCTCTTACGCTGATACGCAC  1020
             |||||||||||||||||||||||||||||||||||||||||||| |||||||| || |||
Sbjct  961   ATCGAGGCATCCCCGGACAAGATGTTGCAAGGTCGTCTCTTCTCCTACGCTGACACTCAC  1020
```

```
Query  1021  CGCCATCGCCTCGGAGCAAACTATCTTCAACTGCCGGTCAACTGCCCGTACCGCGTTGCC  1080
             || ||||| || |||||||||||||||||||||||||| ||||| ||||| |||||| |
Sbjct  1021  CGTCATCGTCTGGGAGCAAACTATCTTCAACTGCCGGTTAACTGTCCGTATCGCGTTTCT  1080

Query  1081  ATGAAGAACTATCAGCGTGATGGCCCGATGAATGTCACCGACAATCAAGGAGGAGCCCCG  1140
             ||||| ||||| || ||||| |||||||||||||| |||||||||||||| || ||||||
Sbjct  1081  ATGAAAAACTACCAACGTGACGGCCCGATGAATGTTACCGACAATCAAGGTGGCGCCCCG  1140

Query  1141  AACTATTATCCCAACTCCTTCGCAGGACCGGAACCGTGCGGATTTGCAAATAAGCTCCAA  1200
             ||||| |||||||| |||||||  ||||| ||||| || ||||||||  ||||||| |||
Sbjct  1141  AACTACTATCCCAATTCCTTCGGTGGACCAGAACCATGTGGATTTGCGCATAAGCTTCAA  1200

Query  1201  AACTCCAAATTCCACGTGTCCGGAGATGTCAACCGGTTCGAGTCCGGCGATACCGAGGAC  1260
             || ||||||||| | || || |||||||| ||||| ||||||||||| || ||||| |||
Sbjct  1201  AATTCCAAATTCAATGTTTCTGGAGATGTAAACCGTTTCGAGTCCGGTGAAACCGAAGAC  1260

Query  1261  AATTTCGCCCAGCCCGCCATTTTCTACCGCCGCGTGCTGGATGAAGCCGCCCGAGAGCGT  1320
             || |||||||| |||| ||||||||| ||||| ||| |||||||||| || || |||||
Sbjct  1261  AACTTCGCCCAACCCGGCATTTTCTATCGCCGTGTGTTGGATGAAGCTGCTCGTGAGCGC  1320

Query  1321  ATGATCACCAACATGGTCAACCACATGTCGGCCGCCTCGTCCTTTATTCAGGAACGCGCC  1380
             ||||||||||||||||| || |||||||| || || ||| |||| ||||| ||||| ||
Sbjct  1321  ATGATCACCAACATGGTTAATCACATGTCCGCAGCTTCGCCCTTCATTCAAGAACGTGCA  1380

Query  1381  GT-CAAGAATTTCTCGCAAGTCGATGCCGATTTTGGACGTCGCTTGACCGAGGGTCTGAA  1439
             || ||| |||||||||||||| ||||| || |||||||| || |||||||||||  ||||
Sbjct  1381  GTTCAA-AATTTCTCGCAAGTTGATGCTGACTTTGGACGCCGTTTGACCGAGGGCTTGAA  1439

Query  1440  GCTACGCCGTTCGGCCAAAATGTAA  1464
              || ||||| || ||||||||||||
Sbjct  1440  ACTGCGCCGCTCAGCCAAAATGTAA  1464
```
