# Supplementary material for: Infections of Aedes Mosquito Cells by Wolbachia Strains wAu and wMelpop Modulate Host Cellular Transcriptomes Differently and Suppress Dengue Viral Replication
Source: Viruses. 2025 Jun 28;17(7):922. doi: 10.3390/v17070922 (PMC12299481; doi:10.3390/v17070922)
Supplement: Supplementary file 1 [file viruses-17-00922-s001.zip › Supplemental table file to viruses 2025.pdf]

The RNA-seq data as raw reads are available as Fastq files in the NCBI Short Read Archive (SRA) Data/Download Web page (The BioProject accession number, PRJNA1117057).

Supplemental Table S1. PCR Primers used for RT-qPCR in measuring virus copy numbers, Wolbachia densities, cellular gene numbers.

PCR fragment	forward	reverse
DENV1	CTTTGGGAGGGATCTCCAGG (7440F)	CTCCCAGTGTTTCCCCTTGG (7640R)
DENV2	CTCAGTGAACGTGTCCCTAAC (7000F)	GAGTCCTGGCCCTATGATGGC (7200R)
DENV3	CAGTACTTTTGCTAGCTACAC (7151f)	GCCCATGATGTTCTCATTAAC (7380R)
DENV4	TGGAACAGAGTGTGGATAGAA (10001F)	CTACAGAAACTCCCTCACTCT (10266R)
16S rRNA	GCGAGGCTAAGCTAATCCCTT (1109F)	AACCAATTCCCATGGCGTGA (1328R)
catalase	GCCGATACCGCTCGTGATCCA (COMMON-F)	CCCAGAACATATCGGCATCCT (COMMON-R)

Supplemental Table S2. Nucleotide and amino acid variations of DENV2 in C6/36 cells, wMelPop-C6/36 cells, and wAu-C6/36 cells at 5 days p.i.

Host cell	C6/36 cells	*w*MelPop-C6/36 cells	*w*Au-C6/36 cells
RNA	DENV2	DENV2	DENV2
virus Collection	supernatant	supernatant	supernatant
5'-UTR	ND	ND	ND
C	ND	ND	ND
prM	ND	ND	ND
E	1399(T155P, 6.2%)<br>1541(E202G, 12.3%)<br>2170(M412L, 6.3%)	1399(T155P, 8.8%)<br>1541(E202G, 10.4%)<br>2170(M412L, 7.1%)	1399(T155P, 7.9%)<br>1837(M301L, 9.9%)<br>2170(M412L, 13.6%)
NS1	ND	ND	ND
NS2A	ND	ND	ND
NS2B	ND	4472(I114T, 8.7%)	4452(S107, silent, 10.2%)
NS3 PRO	ND	ND	ND
NS3 HEL	ND	ND	ND
NS4A-2K	ND	6759 (T128 silent, 7.2%)	6759 (T128 silent, 6.6%)
NS4B	7226(Q134P, 5.6%)	7403(A193D, 7.3%)	ND
NS5 MT	ND	ND	ND
NS5 POL	ND	10251(E894D, 13.2%)	ND
3'-UTR	ND	ND	ND

The cutoff for inclusion is > 5% abundance. The numbers refer to the nucleotide localization (1 to 10723) in DENV2 RNA, followed by the corresponding original amino acid, location, mutated amino acid and frequency of occurrence are shown within parentheses. ND, not detected.

Supplemental Table S3. Nucleotide and amino acid variations of DENV1 in C6/36 cells and *w*MelPop-C6/36 cells at 5 days p.i.

Host cell	C6/36 cells	*w*MelPop-C6/36 cells
RNA	DENV1	DENV1
virus Collection	supernatant	supernatant
5'-UTR	ND	ND
C	ND	ND
prM	673 (T79, silent, 74.9%)	673 (T79, silent, 56.6%)
E	1095 (A56V, 7.8%)<br>1118 (E64K, 84.9%)<br>1302 (T125K, 9.7 %)<br>1416 (T163I, 38.3%)	 1118 (E64K, 64.5%)<br>1302 (T125K, 11.8 %)<br>1416 (T163I, 24.1%)<br>1686 (V253A, 7.8%)
NS1	2803 (V128 silent, 76.8%)	2803 (V128 silent, 60.7%)
NS2A	ND	3778 (V101 silent, 5.3%)
NS2B	ND	ND
NS3PRO	ND	ND
NS3HEL	6022 (T501 silent, 7.7%)	ND
NS4A-2K	ND	ND
2K	ND	ND
NS4B	ND	ND
NS5MT	ND	ND
NS5POL	8494 (S307 silent, 8.4%)<br>9684 (Q704L, 7.3%)	8494 (S307 silent, 6.8%)<br>9684 (Q704L, 5.5%)
3'-UTR	10540 (A to G, 84.0%)	10540 (A to G, 74.2%)

The cutoff for inclusion is > 5% abundance. The numbers refer to the nucleotide localization in the viral RNA, followed by the corresponding original amino acid, location, mutation and frequency of occurrence are shown within parentheses. Bald numbers and letters mean > 50% frequency of amino acid alteration. ND, not detected.

Supplemental Table S4. Nucleotide and amino acid variations of DENV3 in C6/36 cells and *w*MelPop-C6/36 cells at 5 days p.i.

Host cell	C6/36 cells	*w*MelPop-C6/36 cells
RNA	DENV3	DENV3
virus Collection	supernatant	supernatant
5'-UTR	ND	ND
C	ND	ND
prM	455 (D7N, 31.2%)<br>486 (N17S, 23.5%)<br>540 (T35K, 26,6%)<br>575 (D47N, 14.1%)<br>903 (I156T, 27.3%)	455 (D7N, 45.6%)<br>486 (N17S, 33.0%)<br>540 (T35K, 10.4%)<br>575 (D47N, 9.8%)<br>903 (I156T, 38.2%)
E	1491 (R186Q, 25.3%)<br>1570 (F212L 40.4%)<br>2005 (T357 silent, 43.1%)	1491 (R186Q, 34.9%)<br>1570 (F212L 22.0%)<br>2005 (T357 silent, 24.1%)
NS1	3462 (A350V, 38.8%)	3462 (A350V, 17.2%)
NS2A	3642 (I58T, 39.1%)<br>4070 (V201L, 8.8%)	3642 (I58T, 22.9%)
NS2B	ND	ND
NS3PRO	ND	ND
NS3HEL	5303 (A264T, 24.3%)<br>5671 (L386 silent, 27.0%)<br>5916 (L468R, 32.2%)	5303 (A264T, 32.1%)<br>5671 (L386 silent, 37.0%)<br>5916 (L468R, 42.8%)
NS4A-2K	ND	ND
2K	ND	ND
NS4B	ND	ND
NS5MT	8338 (G258 silent, 30.3%)	8338 (G258 silent, 37.6%)
NS5POL	10186 (I874M, 38.4%)	10186 (I874M, 24.6%)
3'-UTR	10318 (A to G, 49.3%)	10318 (A to G, 26.4%)

The cutoff for inclusion is > 5% abundance. The numbers refer to the nucleotide localization in the viral RNA, followed by the corresponding original amino acid, location, mutation and frequency of occurrence are shown within parentheses. Bald numbers and letters mean > 50% frequency of amino acid alteration. ND, not detected.

Supplemental Table S5. Nucleotide and amino acid variations in DENV1 in C6/36 cells and *w*Au-C6/36 cells at 5 days p.i.

Host cell	C6/36 cells	*w*Au-C6/36 cells
RNA	DENV1	DENV1
virus collection	supernatant	supernatant
5'-UTR	ND	ND
C	ND	ND
prM	ND	ND
E	1118 (E64K, 5.7%)<br>1416 (T163I, 13.9%)<br>1538 (E204K, 68.4%)<br>1620 (E231G, 12.4%)<br>1686 (V253A, 6.7%)<br>1764 (T279K, 7.9%)<br>2314 (G462 silent, 13.9%)	1416(T163I, 17.7%)<br>1538 (E204K, 69.9%)<br>1620 (E231G, 6.4%)<br>1686 (V253A, 6.6%)<br>1764 (T279K, 12.8%)<br>2314 (G462 silent, 12.8%)
NS1	2803 (V128 silent, 6.0%)	ND
NS2A	3778 (V101 silent, 5.4%)	3664(N63 silent, 6.7%)<br>3778 (V101 silent, 6.1%)
NS2B	ND	ND
NS3<br>PRO	ND	ND
NS3<br>HEL	6022 (T501 silent, 5.3%)	6022 (T501 silent, 8.4%)
NS4A-2K	ND	ND
NS4B	ND	9215 (Q22P, 8.4%)
NS5MT	ND	ND
NS5POL	10054 (T827 silent, 6.0%)	ND
3'-UTR	10540 (A to G, 5.2%)	ND

The cutoff for inclusion is > 5% abundance. The numbers refer to the nucleotide localization in the viral RNA, followed by the corresponding original amino acid, location, mutation and frequency of occurrence are shown within parentheses. Bald numbers and letters mean > 50% frequency of amino acid alteration. ND, not detected.

Supplemental Table S6. Nucleotide and amino acid variations in DENV3 in C6/36 cells *w*Au-C6/36 cells at 5 days p.i.

Host cell	C6/36 cells	*w*Au-C6/36 cells
RNA	DENV3	DENV3
virus collection	supernatant	supernatant
5'-UTR	ND	ND
C	ND	ND
prM	455 (D7N, 55.3%)<br>486 (N17S, 10.7%)<br>514 (K26N, 7.9%)<br>540 (T34K, 10.0%)<br>727 (P97 silent, 5.9%)<br>903 (I156T, 45.8%)	455 (D7N, 47.0%)<br>486 (N17S, 24.5%)<br>514 (K26N, 9.4%)<br>727 (P97 silent, 8.4%)<br>903 (I156T, 35.5%)
E	1066 (E44 silent, 7.4%)<br>1398 (T155M, 8.6%)<br>1491 (R186Q, 31.0%)<br>1570 (F212L, 12.7%)<br>2005 (T357 silent, 22.2%)	1066 (E44 silent, 10.1%)<br>1393 (N153K, 6.1%)<br>1398 (T155M, 10.7%)<br>1491 (R186Q, 18.6%)<br>1570 (F212L, 6.1%)<br>2005 (T357 silent, 13.0%)
NS1	2669 (L86 silent, 6.0%)<br>3462 (A350V, 12.7%)	2669 (L86 silent, 6.2%)<br>3462 (A350V, 7.4%)
NS2A	3642 (I58T, 14.6%)	3642 (I58T, 5.9%)
NS2B	ND	ND
NS3PRO	ND	ND
NS3HEL	5303 (A95T, 8.5%)<br>5671 (L217 silent, 19.9%)<br>5916 (K299R, 59.6%)<br>6328 (P436 silent, 6.1%)	5303 (A95T, 22.4%)<br>5671 (L217 silent, 35.2%)<br>5916 (K299R, 51.4%)<br>6328 (P436 silent, 7.2%)
NS4A-2K	ND	ND
NS4B	ND	ND
NS5MT	7991 (L143 silent, 5.3%)<br>8338 (G258 silent, 54.7%)	7991 (L143 silent, 6.7%)<br>8338 (G258 silent, 46.0%)
NS5POL	9213 (E550G, 8.0%)<br>10186 (I874M, 13.1%)	10186 (I874M, 5.9%)
3'-UTR	10293 (C to T, 7.5%)<br>10318 (A to G, 20.3%)	10293 (C to T, 9.7%)<br>10318 (A to G, 10.4%)

The cutoff for inclusion is > 5% abundance. The numbers refer to the nucleotide localization in the viral RNA, followed by the corresponding original amino acid, location, mutation and frequency of occurrence are shown within parentheses. Bald numbers and letters mean > 50% frequency of amino acid alteration. ND, not detected.

Supplemental Table S7A. Nucleotide and amino acid variations of DENV2 in Aag2 cells at 5 days p.i.

Host cell	Aag2 cells (1)	Aag2 cells (2)	Aag2DG cells (3)
RNA	DENV2	DENV2	DENV2
virus collection	cell lysis	cell lysis	cell lysis
5’-UTR	ND	ND	ND
C	ND	ND	ND
prM	ND	ND	ND
E	1399 (T155P, 11.7%)<br>1541 (E202G, 32.1%)	1399 (T155P, 12.6%)<br>1541 (E202G, 27.9%)	1399 (T155P, 11.4%)<br>1541 (E202G, 33.3%)
NS1	2451 (N10 silent, 6.6%)	2451 (N10 silent, 6.1%)	2451 (N10 silent, 6.4%)
NS2A	4018 (F181L, 5.8%)<br>4114 (S212 silent, 5.3%)	4018 (F181L, 5.6%)<br>4114 (S212 silent, 5.0%)	4018 (F181L, 5.6%)<br>4114 (S212 silent, 5.5%)
NS2B	4472 (I114T, 22.0%)	4472 (I114T, 18.1%)	4472 (I114T, 23.4%)
NS3<br>PRO	ND	ND	4957 (V146I, 6.4%)
NS3<br>HEL	ND	ND	ND
NS4A-2K	6759 (T128 silent, 10.1%)	6759 (T128 silent, 10.4%)	6759 (T128 silent, 9.7%)
NS4B	ND	ND	ND
NS5MT	ND	ND	ND
NS5POL	10251 (E894D, 26.9%)	8727 (G386 silent, 6.6%)<br>10251 (E894D, 23.4%)	8727 (G386 silent, 6.7%)<br>10251 (E894D, 28.7%)
3’-UTR	ND	ND	ND

Supplemental Table S7B. Nucleotide and amino acid variations in DENV2 in *w*MelPop-Aag2 cells at 5 days p.i.

Host cell	*w*Au-Aag2 cells (1)	*w*Au-Aag2 cells (2)	*w*Au-Aag2 cells (3)
RNA	DENV2	DENV2	DENV2
virus collection	cell lysis	cell lysis	cell lysis
5'-UTR	ND	ND	ND
C	ND	ND	ND
prM	ND	ND	ND
E	1399 (T155P, 11.2%)<br>2170 (M412L, 12.0%)	1399 (T155P, 10.9%)<br>1541 (E202G, 22.5%)	1399 (T155P, 12.1%)<br>1541 (E202G, 9.1%)<br>2170 (M412L, 10.1%)
NS1	ND	ND	ND
NS2A	ND	ND	ND
NS2B	ND	4472 (I114T, 7.8%)	4472 (I114T, 7.3%)
NS3<br>PRO	ND	ND	ND
NS3<br>HEL	ND	ND	ND
NS4A-2K	6759 (T128 silent, 9.7%)	6759 (T128 silent, 5.3%)	6759 (T128 silent, 11.6%)
NS4B	ND	ND	ND
NS5MT	ND	ND	ND
NS5POL	10251 (E894D, 11.2%)	10251 (E894D, 16.7%)	10251 (E894D, 8.5%)
3'-UTR	ND	ND	ND
